# Feasibility and tolerability of eribulin-based chemotherapy versus other chemotherapy regimens for patients with metastatic triple-negative breast cancer: a single-centre retrospective study

**DOI:** 10.3389/fcell.2024.1313610

**Published:** 2024-02-22

**Authors:** Weiwei Huang, Chenxi Wang, Lili Wang, Yangkun Shen, Qi Chen, Zhijian Huang, Jian Liu, Xiaoyan Lin, Fan Wu, Xinhua Chen, Nani Li, Yi Hong, Mulan Chen, Jieyu Li, Chuanzhong Huang

**Affiliations:** ^1^ Department of Medical Oncology, Clinical Oncology School of Fujian Medical University, Fujian Cancer Hospital, Fuzhou, China; ^2^ Department of Medical Oncology, Fujian Medical University Union Hospital, Fuzhou, China; ^3^ Fujian Provincial Key Laboratory of Translational Cancer Medicine, Fuzhou, China; ^4^ Fujian Key Laboratory of Innate Immune Biology, Biomedical Research Center of South China, Fujian Normal University Qishan Campus, College Town, Fuzhou, Fujian Province, China; ^5^ Department of Breast Surgical Oncology, Fujian Cancer Hospital, Fujian Medical University Cancer Hospital, Fuzhou, China; ^6^ Laboratory of Immuno-Oncology, Fujian Cancer Hospital, Fujian Medical University Cancer Hospital, Fuzhou, China

**Keywords:** eribulin, metastatic breast cancer, real-world research, propensity score matching (PSM), efficacy and safety

## Abstract

**Background:** Patients with Triple-negative breast cancer (TNBC) face a poor prognosis and limited therapeutic options. Current data on eribulin usage to treat TNBC is scarce. Therefore, we sought to compare the feasibility and tolerability of eribulin-based regimens with other chemotherapy regimens in patients with TNBC.

**Method:** This retrospective study was conducted at Fujian Medical University Cancer Hospital and included 159 patients with TNBC enrolled between October 2011 and January 2023. Patients underwent treatment with eribulin-based and other chemotherapy regimens. The study’s primary endpoints were progression-free survival (PFS) and overall survival (OS), while its secondary endpoint was objective response rate (ORR), disease control rate (DCR), and safety. Tumour response was assessed using RECIST V.1.1 criteria.

**Results:** Of the 159 participants in the study, 42 individuals (26.4%) received treatment with eribulin, whereas 117 participants (73.6%) were administered alternative chemotherapy regimens, which included nab-paclitaxel-based therapy (n = 45) and platinum-based therapy (n = 51). The follow-up period for all patients ended on 31 December 2022, and the median follow-up time was 18.3 months (range:0.7–27.5). Following propensity score matching (PSM), eribulin-based treatment resulted in longer median progression-free survival compared to platinum-based (hazard ratio (HR) = 0.41, *p* = 0.006), nab-paclitaxel-based (hazard ratio = 0.36, *p* = 0.001) and other chemotherapy (HR = 0.39, *p* < 0.001). Also, eribulin induced a remarkable prolongation of the median overall survival duration in all three comparative groups. The group receiving eribulin treatment showed significantly reduced incidences of any grade of anaemia, peripheral neuropathy, nausea and vomiting, and hair loss compared to other chemotherapy groups.

**Conclusion:** For the salvage treatment of advanced TNBC, treatment with eribulin produced longer median PFS and OS than other chemotherapy regimens, with a well-tolerated safety profile. Therefore, further investigation of eribulin-based treatment in larger randomized trials for patients with advanced TNBC is warranted.

## 1 Introduction

Triple-negative breast cancer (TNBC) accounts for 15%–25% of all breast cancers as it lacks oestrogen receptor (ER), progesterone receptor (PR), and human epidermal growth factor receptor 2 (HER2) expression, and exhibits high genomic instability with a high mutation burden (Harbeck et al., 2019). Currently, the primary treatment modalities for TNBC are surgery and chemotherapy, although these interventions are associated with suboptimal long-term outcomes and higher rates of metastasis and recurrence. Notably, the 5-year survival rate for patients with metastatic TNBC patients is less than 30% (Harbeck et al., 2019). Anthracyclines and taxanes are integral to neoadjuvant and adjuvant chemotherapy for TNBC. Nevertheless, a universal chemotherapy regimen has not yet been established for advanced TNBC patients who have not responded to anthracycline and/or taxane therapy. Traditional chemotherapeutic agents, including epirubicin, gemcitabine, capecitabine, and platinum agents, have demonstrated limited efficacy in TNBC and are associated with severe bone marrow suppression, nausea and vomiting, renal toxicity and hand-foot syndrome. Therefore, the identification of more effective and safer chemotherapy drugs for managing recurrent metastatic TNBC is urgently required (Gradishar et al., 2022).

Eribulin is a synthetic analogue of halichondrin B and a novel microtubule inhibitor, which binds with high affinity to microtubule ends to irreversibly inhibit mitosis, thus leading to cell death (Pereira et al., 2019). Unlike traditional microtubule inhibitors like taxanes, eribulin has a distinct mechanism of action and is therefore effective even in patients resistant to taxanes (Wan et al., 2021). In addition, eribulin exerts non-cytotoxic effects including angiogenesis inhibition, which can enhance drug perfusion in the tumour microenvironment, and reversing the epithelial-mesenchymal transition in tumour cells to suppress tumour invasion and metastasis (Funahashi et al., 2014; Sachdev et al., 2022). Eribulin is approved by the China National Medical Products Administration for use in patients with locally advanced or metastatic breast cancer who have undergone prior anthracycline and taxane chemotherapy (Yuan and Xu, 2021).

In the global phase III EMBRACE trial, eribulin demonstrated a statistically significant improvement in median OS (13.1 vs 10.6 months, *p* = 0.041) and a higher ORR (12% vs 5%, *p* = 0.002) when compared to the treatment selected by the patient’s physician in individuals with recurrent or metastatic breast cancer who had undergone prior treatment with second-to-fifth line chemotherapy (Cortes et al., 2011). The efficacy of eribulin compared to vinorelbine in Chinese patients with locally recurrent or metastatic breast cancer was analysed in a phase III study (Study 304) that was randomized and conducted at multiple centres (Yuan et al., 2019). The results showed a notable improvement in median PFS for eribulin at 3.7 months compared to vinorelbine at 3.1 months, with a PFS ratio of 1.19 (95% confidence interval (CI): 1.03–1.37, *p* = 0.020). Furthermore, eribulin achieved significantly higher ORR of 30.7% compared to vinorelbine’s 16.9%, with *p* < 0.001. However, no statistically significant difference was found with regards to median OS. A sub-analysis of patients diagnosed with TNBC indicated that eribulin had a longer median PFS (2.7 months) compared to vinorelbine (1.4 months), with a hazard ratio (HR) of 0.70 (95% CI: 0.47–1.03). Additionally, the ORR for eribulin was 26.6% compared to vinorelbine’s 14.7%. Safety findings from Study 304 showed that eribulin had a lower incidence of peripheral neuropathy compared to vinorelbine. In Phase III trial (Study 301), eribulin was compared to capecitabine amongst patients suffering from locally advanced or metastatic breast cancer who had undergone prior anthracycline and/or taxane-based therapy (up to second-line for advanced disease) (Kaufman et al., 2015; Twelves et al., 2016; Pivot et al., 2018). Similar survival benefits were observed for Eribulin and capecitabine, with the median OS of patients with TNBC lengthened by 5 months and the median OS of patients with HER2-negative breast cancer extended by 2.6 months. Furthermore, both eribulin and capecitabine had similar incidences regarding treatment-emergent adverse events (TEAEs). Nevertheless, the eribulin group recorded a notably lower incidence of hand-foot syndrome. Analysis of a subgroup of patients with non-visceral metastases showed a significant increase in OS of 9.5 months in the eribulin group compared to capecitabine, with a 49% reduction in the risk of death (Kaufman et al., 2015; Twelves et al., 2016; Pivot et al., 2018).

The above evidence from clinical trials shows that eribulin is effective for the treatment of HER2-negative metastatic breast cancer, with subgroup analyses indicating efficacy against metastatic TNBC. However, since eribulin has only been available in China for a brief period, there is insufficientefficacy and safety data available for Chinese patients with metastatic TNBC. Therefore, a retrospective analysis was undertaken to evaluate the efficacy and safety of eribulin in Chinese patients with metastatic TNBC and compare the clinical outcomes of eribulin-based treatment with other chemotherapy protocols.

## 2 Methods

### 2.1 Patient demographics and outcome measurements

This retrospective study was conducted at a single centre, analysing medical records of adult patients aged 18–70 years with metastatic or unresectable recurrent TNBC(ER/PR-negative and HER2/neu-negative) who underwent chemotherapy at the Fujian Medical University Cancer Hospital between October 2011 and January 2023. In TNBC, ER/PR negativity was defined as ER/PR staining of less than 1%, and HER2/neu negativity was defined as immunohistochemistry (IHC) 0–1+ or IHC 2+ FISH negativity. Measurable lesions, according to the Response Evaluation Criteria in Solid Tumours (RECIST V.1.1), and a minimum of two cycles of chemotherapy were prerequisites for patient inclusion. Eribulin-based treatment was continued until disease progression, unacceptable toxicity, or patient refusal. The primary endpoints were PFS and OS. Secondary endpoint included ORR, DCR, and safety. Tumour response was evaluated using the RECIST V.1.1 criteria. Adverse events (AEs) were recorded and categorized according to the National Cancer Institute Common Terminology Criteria for Adverse Events (NCI-CTCAE 5.0). The study adhered to good clinical practice guidelines and the principles outlined in the Declaration of Helsinki, and all patients provided written informed consent for participation in this study.

### 2.2 Statistical analysis

To reduce the risk of selection bias and other confounding factors, propensity score matching (PSM) was utilised. The PSM model included the following factors: patient age, Eastern Co-operative Oncology Group Performance Score (ECOG PS), menopausal status, prior surgery, TNBC at initial onset, Ki67 status, metastasis site or locations, perioperative treatment, combination therapy type and line of therapy. Matched pairs were then formed using a 1-to-1 nearest-neighbour with a calliper width of 0.2.

Between-group differences were compared using a Student’s t-test or the chi-squared test. OS and PFS were calculated with the Kaplan-Meier method and compared using the log-rank test. Any factors that were statistically significant (*p* < 0.10) in the univariate analysis were candidates for entry into a multivariable Cox proportional-hazards model. All *p*-values were 2-sided, with *p*-values <0.05 considered significant. R version 4.2.3 was used for all statistical analyses.

## 3 Results

### 3.1 Patient characteristics

**FIGURE 1:**
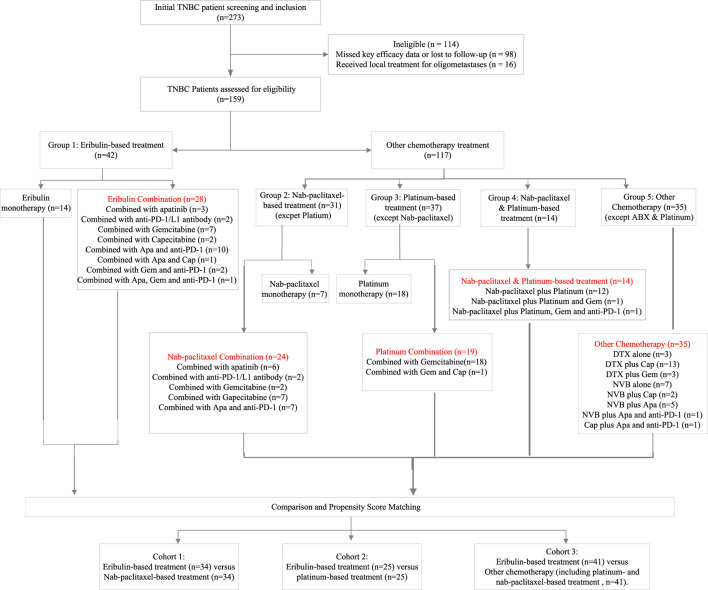
Patient flow chart Apa, apatinib; Cap, capecitabine; DTX, docetaxel; Gem, gemcitabine; TNBC, triple-negative breast cancer.

273 patients with advanced TNBC were screened. 98 patients were excluded due to missing efficacy data or loss to follow-up. Additionally, 16 patients who received local treatment for oligometastases were also excluded. The total number of patients included in the study was 159. Out of this number, 42 patients (26.4%) received eribulin-based treatment which comprised of 14 patients who received single-agent eribulin and 28 patients who received eribulin in combination with other drugs. The remaining 117 patients (73.6%) were treated with other non-eribulin chemotherapy regimens. Of the patients, 45 were administered nab-paclitaxel, while 51 received platinum-based treatments. It is worth noting that 14 of these patients underwent both nab-paclitaxel and platinum-based therapies. In addition, 35 out of the total 117 patients received treatments other than nab-paclitaxel and platinum-based therapies. Based on available patient data, we conducted three comparisons between eribulin-based treatment (n = 42) and nab-paclitaxel-based treatment (n = 45), platinum-based treatment (n = 51), and other types of chemotherapy including nab-paclitaxel- and platinum-based regimens (n = 117). After PSM, there were three comparison groups:eribulin-based treatment (n = 34) *versus* nab-paclitaxel-based treatment (n = 34), eribulin-based treatment (n = 25) *versus* platinum-based treatment (n = 25) and eribulin-based treatment (n = 41) *versus* other chemotherapy (including platinum- and nab-paclitaxel-based regimens, n = 41). The treatment details are outlined in Figure 1. Baseline and disease characteristics before and after PSM for the comparison of eribulin-based treatment *versus* nab-paclitaxel-based treatment, eribulin-based treatment *versus* platinum-based treatment and eribulin-based treatment *versus* other chemotherapy groups are shown in Tables 1–3.

**TABLE 1:** Patient baseline demographic and clinical characteristics in the eribulin-based and NAB-paclitaxel-based groups.

	Initial cohort			Propensity-score-matched cohort		
	Eribulin based (n = 42)	Nab-paclitaxel based (n = 45)	*p*	Eribulin based (n = 34)	Nab-paclitaxel based (n = 34)	*p*
Age, years						
<50	21 (50.0)	20 (44.4)	*0.761*	15 (44.1)	14 (41.2)	*1.000*
≥50	21 (50.0)	25 (55.6)		19 (55.9)	20 (58.8)	
ECOG PS at start						
0	7 (16.7)	4 (8.9)	*0.442*	4 (11.8)	4 (11.8)	*1.000*
≥1	35 (83.3)	41 (91.1)		30 (88.2)	30 (88.2)	
Menopausal status at diagnosis						
Premenopausal	35 (83.3)	33 (73.3)	*0.385*	27 (79.4)	25 (73.5)	*0.775*
Postmenopausal	7 (16.7)	12 (26.7)		7 (20.6)	9 (26.5)	
Surgery on primary tumor						
Yes	32 (76.2)	37 (82.2)	*0.668*	27 (79.4)	27 (79.4)	*1.000*
No	10 (23.8)	8 (17.8)		7 (20.6)	7 (20.6)	
TNBC at the initial onset						
Yes	31 (73.8)	36 (80.0)	*0.667*	26 (76.5)	26 (76.5)	*1.000*
No	11 (26.2)	9 (20.0)		8 (23.5)	8 (23.5)	
Ki67 ≥ 30%	38 (90.5)	40 (88.9)	*1.000*	30 (88.2)	29 (85.3)	*1.000*
Metastatic sites						
Visceral	24 (57.1)	23 (51.1)	*0.727*	16 (47.1)	17 (50.0)	*1.000*
Non-visceral	18 (42.9)	22 (48.9)		18 (52.9)	17 (50.0)	
Metastatic sites >3	15 (35.7)	18 (40.0)	*0.849*	12 (35.3)	11 (32.4)	*1.000*
Location of metastases						
Brain	1 (2.4)	1 (2.2)	*1.000*	1 (2.9)	1 (2.9)	*1.000*
Bone	16 (38.1)	20 (44.4)	*0.702*	14 (41.2)	12 (35.3)	*0.803*
Liver	9 (21.4)	8 (17.8)	*0.874*	7 (20.6)	8 (23.5)	*1.000*
Lung	19 (45.2)	20 (44.4)	*1.000*	13 (38.2)	14 (41.2)	*1.000*
Lymph node	30 (71.4)	31 (68.9)	*0.981*	25 (73.5)	22 (64.7)	*0.600*
Adrenal glands	2 (4.8)	0	*0.444*	0	0	*NA*
Chest wall	7 (16.7)	7 (15.6)	*1.000*	4 (11.8)	4 (11.8)	*1.000*
(Neo-) Adjuvant therapies						
Paclitaxel/Docetaxel	30 (71.4)	29 (64.4)	*0.640*	25 (73.5)	23 (67.6)	*0.790*
Paclitaxel/Docetaxel and Anthracyclines	30 (71.4)	32 (71.1)	*1.000*	9 (26.5)	8 (23.5)	*1.000*
Platinum (Cis/Carbo)	3 (7.1)	1 (2.2)	*0.560*	2 (5.9)	1 (2.9)	*1.000*
Capecitabine	8 (19.0)	4 (8.9)	*0.288*	6 (17.6)	3 (8.8)	*0.474*
Treatment						
Apatinib	15 (35.7)	13 (28.9)	*0.652*	13 (38.2)	11 (32.4)	*0.800*
anti-PD-1/L1 antibody	15 (35.7)	10 (22.2)	*0.249*	13 (38.2)	8 (23.5)	*0.294*
Gemcitabine	10 (23.8)	4 (8.9)	*0.109*	7 (20.6)	4 (11.8)	*0.510*
Capecitabine	3 (7.1)	7 (15.6)	*0.372*	3 (8.8)	3 (8.8)	*1.000*
Lines of therapy						
1st Line	17 (40.5)	20 (44.4)	*0.875*	13 (38.2)	12 (35.3)	*1.000*
2nd^+^ Line	25 (59.5)	25 (55.6)		21 (61.8)	22 (64.7)	

**TABLE 2:** Patient baseline demographic and clinical characteristics in eribulin-based and platinum-based groups.

	Initial cohort			Propensity-score-matched cohort		
	Eribulin based (n = 42)	Platinum based (n = 51)	*p*	Eribulin based (n = 25)	Platinum based (n = 25)	*p*
Age, years						
<50	21 (50.0)	26 (51.0)	*1.000*	10 (40.0)	10 (40.0)	*1.000*
≥50	21 (50.0)	25 (49.0)		15 (60.0)	15 (60.0)	
ECOG PS at start						
0	7 (16.7)	5 (9.8)	*0.502*	5 (20.0)	4 (16.0)	*1.000*
≥1	35 (83.3)	46 (90.2)		20 (80.0)	21 (84.0)	
Menopausal status at diagnosis						
Premenopausal	35 (83.3)	38 (74.5)	*0.437*	20 (80.0)	19 (76.0)	*1.000*
Postmenopausal	7 (16.7)	13 (25.5)		5 (20.0)	6 (24.0)	
Surgery on primary tumor						
Yes	32 (76.2)	36 (70.6)	*0.710*	18 (72.0)	19 (76.0)	*1.000*
No	10 (23.8)	15 (29.4)		7 (28.0)	6 (24.0)	
TNBC at the initial onset						
Yes	31 (73.8)	42 (82.4)	*0.457*	19 (76.0)	19 (76.0)	*1.000*
No	11 (26.2)	9 (17.6)		6 (24.0)	6 (24.0)	
Ki67 ≥ 30%	38 (90.5)	44 (86.3)	*0.763*	22 (88.0)	23 (92.0)	*1.000*
Metastatic sites						
Visceral	24 (57.1)	30 (58.8)	*1.000*	13 (52.0)	16 (64.0)	*0.567*
Non-visceral	18 (42.9)	21 (41.2)		12 (48.0)	9 (36.0)	
Metastatic sites >3	15 (35.7)	26 (51.0)	*0.206*	9 (36.0)	11 (44.0)	*0.773*
Location of metastases						
Brain	1 (2.4)	1 (2.0)	*1.000*	0	0	*1.000*
Bone	16 (38.1)	21 (41.2)	*0.929*	5 (20.0)	11 (44.0)	*0.330*
Liver	9 (21.4)	9 (17.6)	*0.845*	5 (20.0)	5 (20.0)	*1.000*
Lung	19 (45.2)	23 (45.1)	*1.000*	12 (48.0)	14 (56.0)	*0.777*
Lymph node	30 (71.4)	36 (70.6)	*1.000*	17 (68.0)	17 (68.0)	*1.000*
Adrenal glands	2 (4.8)	0	*0.391*	0	0	*1.000*
Chest wall	7 (16.7)	6 (11.8)	*0.705*	2 (20.0)	6 (24.0)	*1.000*
(Neo-) Adjuvant therapies						
Paclitaxel/Docetaxel	30 (71.4)	34 (66.7)	*0.788*	8 (32.0)	7 (28.0)	*1.000*
Paclitaxel/Docetaxel and Anthracyclines	30 (71.4)	40 (78.4)	*0.591*	17 (68.0)	18 (72.0)	*1.000*
Platinum (Cis/Carbo)	3 (7.1)	1 (2.0)	*0.476*	2 (8.0)	0	*0.470*
Capecitabine	8 (19.0)	4 (7.8)	*0.196*	2 (8.0)	3 (12.0)	*1.000*
Treatment						
Apatinib	15 (35.7)	0	*<0.001*	0	0	*NA*
anti-PD-1/L1 antibody	15 (35.7)	1 (2.0)	*<0.001*	4 (16.0)	1 (4.0)	*0.346*
Gemcitabine	10 (23.8)	21 (41.2)	*0.122*	9 (36.0)	8 (32.0)	*1.000*
Capecitabine	3 (7.1)	1 (2.0)	*0.476*	2 (8.0)	0	*0.470*
Lines of therapy						
1st Line	17 (40.5)	19 (37.3)	*0.918*	9 (36.0)	8 (32.0)	*1.000*
2nd^+^ Line	25 (59.5)	32 (62.7)		16 (64.0)	17 (68.0)	

**TABLE 3:** Patient baseline demographic and clinical characteristics in eribulin-based and other chemotherapy groups.

	Initial cohort			Propensity-score-matched cohort		
	Eribulin based (n = 42)	Other chemotherapy (n = 117)	*p*	Eribulin based (n = 41)	Other chemotherapy (n = 41)	*p*
Age, years						
<50	21 (50.0)	54 (46.2)	*0.804*	21 (51.2)	22 (53.7)	*1.000*
≥50	21 (50.0)	63 (53.8)		20 (48.8)	19 (46.3)	
ECOG PS at start						
0	7 (16.7)	9 (7.7)	*0.174*	6 (14.6)	5 (12.2)	*1.000*
≥1	35 (83.3)	108 (92.3)		35 (85.4)	36 (87.8)	
Menopausal status at diagnosis						
Premenopausal	35 (83.3)	85 (72.6)	*0.241*	34 (82.9)	31 (75.6)	*0.586*
Postmenopausal	7 (16.7)	32 (28.2)		7 (17.1)	10 (24.4)	
Surgery on primary tumor						
Yes	32 (76.2)	84 (71.8)	*0.728*	31 (75.6)	29 (70.7)	*0.803*
No	10 (23.8)	33 (28.2)		10 (24.4)	12 (29.3)	
TNBC at the initial onset						
Yes	31 (73.8)	89 (76.1)	*0.934*	31 (75.6)	32 (78.0)	*1.000*
No	11 (26.2)	28 (23.9)		10 (24.4)	9 (22.0)	
Ki67 ≥ 30%	38 (90.5)	102 (87.2)	*0.774*	37 (70.2)	40 (97.6)	*0.356*
Metastatic sites						
Visceral	24 (57.1)	62 (53.0)	*0.777*	23 (56.1)	21 (51.2)	*0.825*
Non-visceral	18 (42.9)	55 (47.0)		18 (43.9)	20 (48.8)	
Metastatic sites >3	15 (35.7)	56 (47.9)	*0.239*	14 (34.1)	17 (41.5)	*0.649*
Location of metastases						
Brain	1 (2.4)	5 (4.3)	*0.936*	1 (2.4)	1 (2.4)	*1.000*
Bone	16 (38.1)	51 (43.6)	*0.663*	16 (39.0)	17 (41.5)	
Liver	9 (21.4)	23 (19.7)	*0.983*	9 (22.0)	7 (17.1)	*0.781*
Lung	19 (45.2)	47 (40.2)	*0.697*	22 (53.7)	23 (56.1)	*1.000*
Lymph node	30 (71.4)	83 (70.9)	*1.000*	29 (70.7)	32 (78.0)	*0.613*
Adrenal glands	2 (4.8)	2 (1.7)	*0.611*	1 (2.4)	2 (4.9)	*1.000*
Chest wall	7 (16.7)	14 (12.0)	*0.613*	6 (14.6)	6 (14.6)	*1.000*
(Neo-) Adjuvant therapies						
Paclitaxel/Docetaxel	30 (71.4)	74 (63.2)	*0.443*	29 (70.7)	26 (63.4)	*0.638*
Paclitaxel/Docetaxel and Anthracyclines	30 (71.4)	83 (70.9)	*1.000*	29 (70.7)	29 (70.7)	*1.000*
Platinum (Cis/Carbo)	3 (7.1)	5 (4.3)	*0.750*	3 (7.3)	3 (7.3)	*1.000*
Capecitabine	8 (19.0)	7 (6.0)	*0.029*	8 (19.5)	3 (7.3)	*0.195*
Treatment						
Apatinib	15 (35.7)	20 (17.1)	*0.023*	15 (36.6)	16 (39.0)	*1.000*
anti-PD-1/L1 antibody	15 (35.7)	12 (10.2)	*<0.001*	15 (36.6)	8 (19.5)	*0.140*
Gemcitabine	10 (23.8)	26 (22.2)	*1.000*	10 (24.4)	10 (24.4)	*1.000*
Capecitabine	3 (7.1)	24 (20.5)	*0.082*	3 (7.3)	5 (12.2)	*0.710*
Lines of therapy						
1st Line	17 (40.5)	49 (41.9)	*1.000*	16 (39.0)	174 (41.5)	*1.000*
2nd^+^ Line	25 (59.5)	68 (58.1)				

### 3.2 Treatment effectiveness

#### 3.2.1 Eribulin-based *versus* nab-paclitaxel-based therapy

**FIGURE 2:**
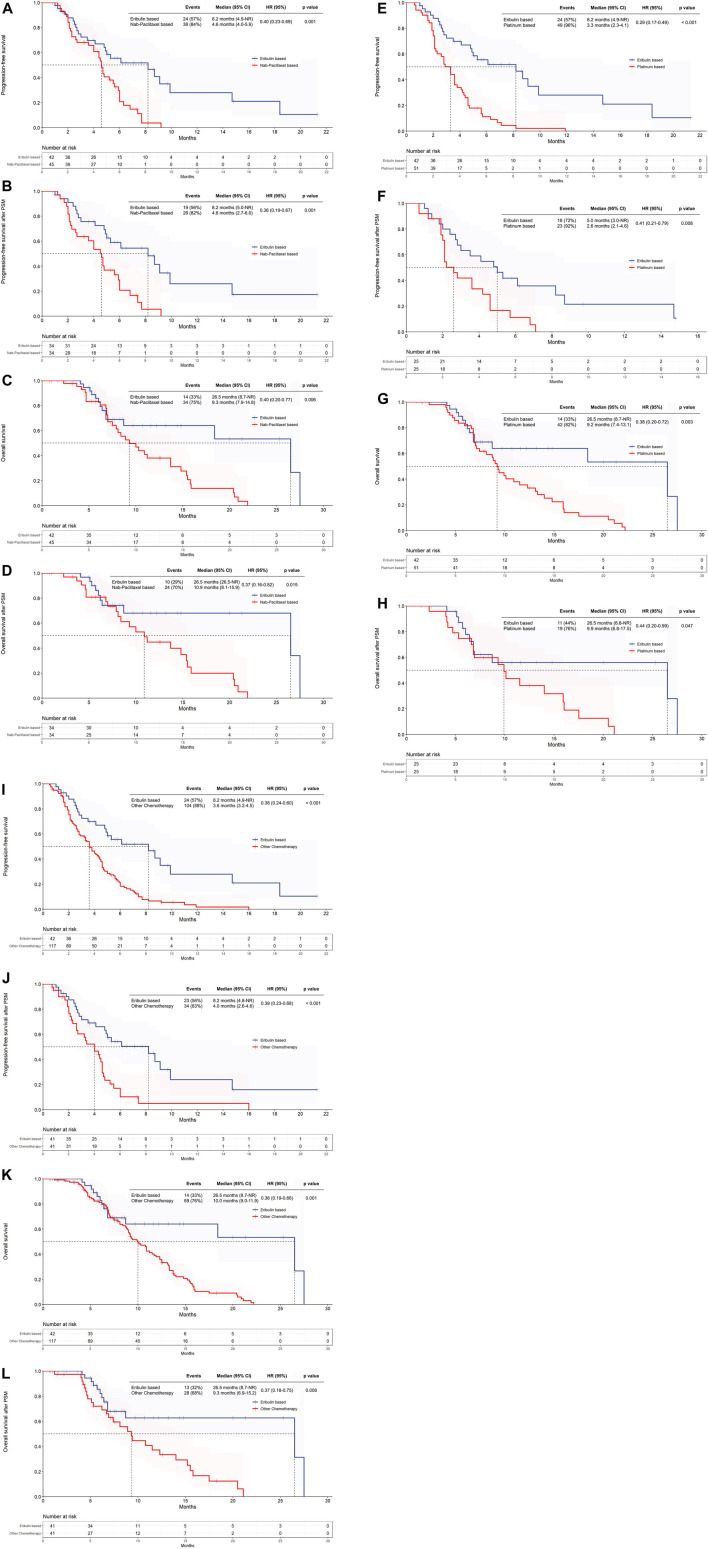
Kaplan-Meier Analysis of Survival for Eribulin and Nab-paclitaxel based group showing progression-free survival **(A)** and overall survival **(B)** in the initial cohort, and progression-free survival **(C)** and overall survival **(D)** in the propensity-score-matched cohort. Kaplan-Meier Analysis of Survival for Eribulin and Platinum based group showing progression-free survival **(E)** and overall survival **(F)** in the initial cohort, and progression-free survival **(G)** and overall survival **(H)** in the propensity-score-matched cohort. Kaplan-Meier Analysis of Survival for Eribulin and other chemotherapy group. Kaplan-Meier curves for progression-free survival **(I)** and overall survival **(J)** in the initial cohort, and progression-free survival **(K)** and overall survival **(L)** in the propensity-score-matched cohort.

In the overall population of patients with advanced TNBC receiving eribulin-based treatment, the ORR and DCR were recorded at 50.0% and 64.3%, respectively. In patients who received first-line treatment, the ORR and DCR were observed at 64.7% and 76.5%, respectively, while in those who had received second-line treatment or beyond, the ORR and DCR were reported at 40.0% and 56.0%.Comparison of eribulin-based treatment to nab-paclitaxel-based treatment in all patients suggested some advantages in terms of ORR and DCR, but the differences were not statistically significant (Table 4). Patients receiving eribulin-based treatment demonstrated a significant improvement in PFS compared to those receiving nab-paclitaxel-based treatment (8.2 vs. 4.6 months, HR = 0.40, *p* = 0.001) (Figure 2A). Furthermore, those receiving eribulin-based treatment had a longer median OS than those receiving nab-paclitaxel-based treatment (26.5 vs. 9.3 months, HR = 0.40, *p* = 0.006) (Figure 2B).

**TABLE 4:** Tumor response per RECIST 1.1 (before PSM).

	Eribulin based	Nab-paclitaxel based	*p*	Platinum based	*p*	Other chemotherapy	*p*
All comers, n	42	45	*-*	51	*-*	117	*-*
ORR, n (%; 95% CI)	21 (50.0; 34.2–65.8)	18 (40.0; 25.7–55.7)	*0.349*	14 (27.5; 15.9–41.7)	*0.026*	37 (31.6; 23.3–40.9)	*0.034*
DCR, n (%; 95% CI)	27 (64.3; 48.0–78.4)	23 (51.1; 35.8–66.3)	*0.214*	26 (51.0; 36.6–65.2)	*0.197*	61 (52.1; 42.7–61.5)	*0.174*
Overall response, n (%)							
Complete response	0	0		0		0	
Partial response	21 (50.0)	18 (40.0)		14 (27.5)		37 (31.6)	
Stable disease	6 (14.3)	5 (11.1)		12 (23.5)		24 (20.5)	
Progressive disease	15 (35.7)	20 (44.4)		24 (47.1)		52 (44.4)	
Not evaluable	0	2 (4.4)		1 (2.0)		4 (3.4)	
1st line, n	17	20	*-*	19	*-*	49	*-*
ORR, n (%; 95% CI)	11 (64.7; 38.3–85.8)	9 (45.0; 23.1–68.5)	*0.456*	6 (31.6; 12.6–56.6)	*0.262*	19 (38.8; 25.2–53.8)	*0.064*
DCR, n (%; 95% CI)	13 (76.5; 50.1–93.2)	12 (60.0; 36.1–80.9)	*-*	11 (57.9; 33.5–79.7)	*-*	30 (61.2; 46.2–74.8)	*-*
Overall response, n (%)							
Complete response	0	0		0		0	
Partial response	11 (64.7)	9 (45.0)		6 (31.6)		19 (38.8)	
Stable disease	2 (11.8)	3 (15.0)		5 (26.3)		11 (22.4)	
Progressive disease	4 (23.5)	6 (30.0)		7 (36.8)		15 (30.6)	
Not evaluable	0	2 (10.0)		1 (5.3)		4 (8.2)	
2nd^+^ line, n	25	25	*-*	32	*-*	68	*-*
ORR, n (%; 95% CI)	10 (40.0; 21.1–61.3)	9 (36.0; 18.0–57.5)	*0.771*	8 (25.0; 11.5–43.4)	*0.227*	18 (26.4; 16.5–38.6)	*0.207*
DCR, n (%; 95% CI)	14 (56.0; 34.9–75.6)	11 (44.0; 24.4–65.1)	*-*	15 (46.9; 29.1–65.3)	*-*	31 (45.6; 33.5–58.1)	*-*
Overall response, n (%)							
Complete response	0	0		0		0	
Partial response	10 (40.0)	9 (36.0)		8 (25.0)		18 (26.4)	
Stable disease	4 (16.0)	2 (8.0)		7 (21.9)		13 (19.1)	
Progressive disease	11 (44.0)	14 (56.0)		17 (53.1)		37 (54.4)	
Not evaluable	0	0		0		0	

After PSM, the eribulin-based treatment group had a longer median PFS compared to the nab-paclitaxel-based group: 8.2 *versus* 4.6 months, HR = 0.36, *p* = 0.001 (Figure 2C). Additionally, the median OS was longer with eribulin compared to nab-paclitaxel: 26.5 *versus* 10.9 months, HR = 0.37, *p* = 0.015 (Figure 2D). Table 5 shows the response rates after PSM. The results of the multivariate analysis showed that the type of treatment (eribulin vs nab-paclitaxel, HR = 0.39, *p* = 0.003) and apatinib-based combination therapy (HR = 0.43, *p* = 0.019) were independent predictors of extended PFS. Furthermore, the type of treatment (eribulin vs nab-paclitaxel, HR = 0.39, *p* = 0.023) was also an independent predictor for longer OS (Supplementary Figure S1).

**TABLE 5:** Tumor response per RECIST 1.1 (after PSM).

	Eribulin based	Nab-paclitaxel based	*p*	Eribulin based	Platinum based	*p*	Eribulin based	Other chemotherapy	*p*
All comers, n	34	34		25	25	*-*	41	41	*-*
ORR, n (%; 95% CI)	17 (50.0; 32.4–67.6))	14 (41.2; 24.6–59.3)	*0.626*	9 (36.0; 18.0–57.5)	5 (20.0; 6.8–40.7)	*0.208*	20 (48.8; 32.9–64.9)	15 (36.6; 22.1–53.1)	*0.264*
DCR, n (%; 95% CI)	23 (67.6; 49.5–82.6)	19 (55.9; 37.9–72.8)	*0.454*	12 (48.0; 27.8–68.7)	12 (48.0; 27.8–68.7)	*1.000*	26 (63.4; 46.9–77.9)	23 (56.1; 39.7–71.5)	*0.499*
Overall response, n (%)									
Complete response	0	0		0	0		0	0	
Partial response	17 (50.0)	14 (41.2)		9 (36.0)	5 (20.0)		20 (48.8)	15 (36.6)	
Stable disease	6 (17.6)	5 (14.7)		3 (12.0)	7 (28.0)		6 (14.6)	8 (19.5)	
Progressive disease	11 (32.4)	14 (41.2)		13 (52.0)	13 (52.0)		15 (36.6)	16 (39.0)	
Not evaluable	0	1 (2.9)		0	0		0	2 (49)	
1st line, n	13	12	*-*	9	8	*-*	16	17	*-*
ORR, n (%; 95% CI)	9 (69.2; 38.6–90.9)	5 (41.7; 15.2–72.3)	*0.333*	4 (44.4; 13.7–78.8)	2 (25.0; 3.2–65.1)	*0.439*	10 (62.5; 35.4–84.8)	8 (47.1; 23.0–72.2)	*0.544*
DCR, n (%; 95% CI)	11 (84.6; 54.6–98.1)	8 (66.7; 34.9–90.1)	*-*	6 (66.7; 30.0–92.5)	5 (62.5; 24.5–91.5)	*-*	12 (75.0; 47.6–92.7)	11 (64.7; 38.3–85.8)	*-*
Overall response, n (%)									
Complete response	0	0		0	0		0	0	
Partial response	9 (69.2)	5 (41.7)		4 (44.4)	2 (25.0)		10 (62.5)	8 (47.1)	
Stable disease	2 (15.4)	3 (25.0)		2 (22.2)	3 (37.5)		2 (12.5)	3 (17.6)	
Progressive disease	2 (15.4)	3 (25.0)		3 (33.3)	3 (37.5)		4 (25.0)	4 (23.5)	
Not evaluable	0	1 (8.3)		0	0		0	2 (11.8)	
2nd^+^ line, n	21	22	*-*	16	17	*-*	25	24	*-*
ORR, n (%; 95% CI)	8 (38.1; 18.1–61.6)	9 (40.9; 20.7–63.6)	*0.850*	5 (31.3; 11.0–58.7)	3 (17.6; 3.8–43.4)	*0.482*	10 (40.0; 21.1–61.3)	7 (29.2; 12.6–51.1)	*0.426*
DCR, n (%; 95% CI)	12 (57.1; 34.0–78.2)	11 (50.0; 28.2–71.8)	*-*	6 (37.5; 15.2–64.6)	7 (41.2; 18.4–67.1)	*-*	14 (56.0; 34.9–75.6)	12 (50.0; 29.1–70.9)	*-*
Overall response, n (%)									
Complete response	0	0		0	0		0	0	
Partial response	8 (38.1)	9 (40.9)		5 (31.3)	3 (17.7)		10 (40.0)	7 (29.2)	
Stable disease	4 (19.0)	2 (9.1)		1 (6.2)	4 (23.5)		4 (16.0)	5 (20.8)	
Progressive disease	9 (42.9)	11 (50.0)		10 (62.5)	10 (58.8)		11 (44.0)	12 (50.0)	
Not evaluable	0	0		0	0		0	0	

#### 3.2.2 Eribulin-based *versus* platinum-based treatment

The ORR and DCR were higher for patients treated with eribulin than platinum-based therapy, both in the entire population and in first-line and second-line or later settings (Table 4). Compared to platinum-based therapy, eribulin-based treatment resulted in a longer median PFS (8.2 vs 3.3 months, HR = 0.29, *p* < 0.001) and a longer median overall survival (OS) (26.5 vs 9.2 months, HR = 0.38, *p* = 0.003) (Figures 2E, F).

After PSM, both the eribulin and platinum-based therapy groups were compared, revealing a longer median PFS in the eribulin group: 5.0 *versus* 2.6 months, HR = 0.41, *p* = 0.008 (Figure 2G). Additionally, the eribulin group showed a longer median OS compared to the platinum-based therapy group: 26.5 *versus* 9.9 months, HR = 0.44, *p* = 0.047 (Figure 2H). The findings detailing the univariate and multivariate analyses for PFS and OS can be observed in Supplementary Figure S2. The type of treatment administered (eribulin vs platinum, HR = 0.49, *p* = 0.039) and being under the age of 50 (HR = 0.45, *p* = 0.016) were identified to be independent determinants of longer PFS.

#### 3.2.3 Eribulin-based *versus* other chemotherapy regimens

Eribulin-based treatment resulted innumerically higher ORR and DCR as compared to other chemotherapy treatments, in the entire population, as well as in first-line treatment, and second-line or subsequent treatments (Table 4). PFS was significantly enhanced with eribulin-based treatment in comparison to others: 8.2 *versus* 3.6 months, respectively, HR = 0.38, *p* < 0.001 (Figure 2I). Meanwhile, median OS was also found to be improved with this treatment: 26.5 compared to 10.0 months, HR = 0.36, *p* = 0.001 (Figure 2J).

After PSM, patients treated with eribulin had extended median PFS (8.2 vs 4.0 months, HR = 0.39, *p* < 0.001) and median OS (26.5 vs 9.3 months, HR = 0.37, *p* = 0.006) compared to those who received alternative chemotherapy regimens (Figures 2K, L). The results of the multivariate analysis show that treatment type (eribulin vs other chemotherapy, HR = 0.30, *p* = 0.001), the presence of visceral metastasis (HR = 1.99, *p* = 0.015), and combination therapy with apatinib (HR = 0.28, *p* = 0.001) were independent predictors of longer PFS. Treatment type (eribulin vs other chemotherapy, HR = 0.47, *p* = 0.047) and ECOG score (0 vs one to two, HR = 0.27, *p* = 0.042) were independent predictors of longer OS (Supplementary Figure S3.

### 3.3 Safety

Overall, eribulin was well tolerated. The hematological adverse events (AEs) observed included neutropenia in 81.0% of cases, anaemia in 35.7%, and thrombocytopenia in 16.7%. Among the non-hematological toxicities, elevated alanine aminotransferase (ALT) levels were the most prevalent at 38.1%, followed by fatigue at 35.7%, hair loss at 33.3%, hand-foot syndrome at 23.8%, peripheral neuropathy at 11.9%, and nausea and vomiting at 7.1% (Supplementary Table S1). Neutropenia (26.2%) was the most frequent Grade 3/4 hematologic AEs, while thrombocytopenia (2.4%) occurred less frequently. No cases of Grade 3/4 anaemia were recorded. Only elevated ALT levels (7.1%) and fatigue (2.4%) were the non-hematological AEs observed in Grade 3/4. No Grade 3/4 peripheral neuropathy, nausea, vomiting, diarrhoea or hand-foot syndrome were observed (Supplementary Table S2). In comparison to chemotherapy regimens based on platinum, eribulin-based chemotherapy exhibited notably lower occurrences of peripheral neuropathy, nausea and vomiting, and hair loss of any grade. Additionally, when compared to other chemotherapy regimens featuring platinum and nab-paclitaxel, eribulin-based chemotherapy demonstrated significantly lower rates of any grade anaemia, peripheral neuropathy, nausea and vomiting, and hair loss (Supplementary Table S1).

## 4 Discussion

In this study, eribulin-based treatment achieved an ORR of 50.0% and a DCR of 64.3% in the whole patient population, with rates of 64.7% and 76.5% when used in first-line, and 40.0% and 56.0% in second-line and subsequent lines, respectively (Table 4). The median PFS for the entire group treated with eribulin was 8.2 months, and the median OS was 26.5 months (Figures 2A, B). In general, these outcomes are consistent with or superior to those reported by previous studies of eribulin. For example, a retrospective study conducted earlier involved 513 patients with metastatic breast cancer, comprising 49.9% of TNBC patients, who were administered eribulin between 1 January 2011, and 31 December 2017. The majority of patients (78.0%) were in the third-line, while the remaining patients were in the fourth-line or beyond. For patients diagnosed with TNBC in this study, the ORR was found to be 55.1%, the median PFS was 5.8 months (95% CI: 5.1–6.4), and the median OS was 9.8 months (95% CI: 8.6–11.0) (Mougalian et al., 2021). The ESEMPiO study retrospectively analyzed data on 574 patients with metastatic breast cancer treated with eribulin between 2012 and 2014. Among 70 patients with TNBC, the median OS and PFS were 9.1 months (95% CI: 5.3–13.4) and 2.8 months (95% CI: 2.3–3.4), respectively (Barni et al., 2019). The longer PFS and OS observed in our study may be attributed to several factors. Firstly, a significant proportion (40.5%) of patients in our study received eribulin as their primary treatment. It has been demonstrated that initial use of eribulin is also associated with a greater PFS benefit than second-line use (Inoue et al., 2020). Secondly, the favourable tolerability and safety of eribulin enable its combination with other chemotherapy drugs to attain improved response rates and survival (Park et al., 2017; Perez−Garcia and Cortes, 2019). The research reveals that a noteworthy proportion of patients who received eribulin treatment were able to undergo combination therapy. Of the 42 patients who underwent eribulin treatment, 28 patients (66.6%) were administered eribulin in combination with other drugs, including chemotherapeutic agents. Thirdly, combining eribulin with anti-angiogenic drugs may enhance treatment outcomes. Patients with TNBC present significantly higher VEGF expression compared to those without TNBC, and anti-angiogenic drugs such as bevacizumab and apatinib exhibit efficacy against TNBC (Zhao et al., 2020). The RIBBON-1 phase III trial demonstrated that addition of bevacizumab to standard therapy with capecitabine or anthracycline/taxane regimens significantly enhanced the median PFS and slightly improved the median OS for patients with locally recurrent or metastatic TNBC. In addition, the treatment was well-tolerated. (Cameron et al., 2013; Miller et al., 2018). Furthermore, in a phase II study, a regimen consisting of eribulin 1.4 mg/m^2^ (on days 1 and 8), camrelizumab 200 mg (on day 1) and apatinib 250 mg once daily every 21 days resulted in an ORR of 37.0%, DCR of 87.0% and a median PFS of up to 8.1 months in 46 patients with advanced TNBC, which was superior to the standard of care in advanced TNBC (Liu et al., 2022). In our study, a greater percentage of individuals in the eribulin group were administered apatinib compared to the other chemotherapy group (35.7% vs 17.1%, *p* = 0.023) (Table 3). The finding imply that eribulin treatment combined with apatinib-based therapy may have led to enhanced survival rates for patients. Finally, eribulin was combined with anti-PD-1 antibodies in 15 patients (35.7%), a significantly higher proportion than the other chemotherapy group who received anti-PD-1 antibodies (10.2%) (Table 3). Despite ongoing debate regarding the value of using anti-PD-1 antibodies for recurrent and metastatic TNBC after multiple lines of treatment, the combination of eribulin and anti-PD-1 antibodies may have improved survival.

To reduce the possibility of selection bias and other confounding factors, we used propensity score matching (PSM). Our study found that after applying PSM, patients treated with eribulin had some advantages in ORR and DCR compared with those treated with other chemotherapies, both in the overall population and in the first- and second-line and above populations. However, no statistically significant difference was seen (Table 5). The retrospective nature of our study and the limited sample size may have contributed to this result. There was a prolongation of PFS and OS in the eribulin-treated group compared to patients receiving other forms of chemotherapy. PFS increased from 4.0 to 8.2 months (HR = 0.39, *p* < 0.001), while OS increased from 9.3 to 26.5 months (HR = 0.37, *p* = 0.006) (Figures 2K, L). Our findings are consistent with previous studies of HER2-negative breast cancer patients, including those with triple-negative breast cancer, who were treated with the eribulin regimen. This treatment has shown superior benefits for progression-free survival (PFS) and overall survival (OS) compared to other chemotherapy regimens (Cortes et al., 2011; Kaufman et al., 2015; Twelves et al., 2016; Pivot et al., 2018; Yuan et al., 2019). The multivariate analyses results indicated risk factors for an association with PFS or OS after PSM between eribulin and other chemotherapy groups. The results reveal that the type of treatment (eribulin vs other chemotherapy), the existence of visceral metastasis, and the usage of apatinib were autonomous predictors of PFS. Additionally, the type of treatment (eribulin vs other chemotherapy) and baseline ECOG score (0 vs 1–2) were independent predictive factors for OS (Supplementary Figure S3). This result suggests that eribulin-based treatment can provide benefits in both PFS and OS for patients with advanced TNBC. Patients who possess a satisfactory ECOG score may have less tumour burden and better physical condition, which may allow them to tolerate stronger combination regimens with eribulin and therefore achieve a longer OS.

Platinum-based chemotherapy has demonstrated significant efficacy in treating TNBC, with particular benefits seen in patients who have inherited germline BRCA1/2 mutations, making platinum a preferred clinical option. Results from the phase II TBCRC009 trial indicated that single-agent cisplatin and carboplatin showed ORRs of 32.6% and 25.6%, respectively, when used to treat patients with metastatic TNBC (Isakoff et al., 2015). The TNT study also compared the efficacy of single-agent carboplatin and docetaxel in unselected patients with advanced TNBC and reported similar ORR, median PFS, and OS for both agents. Notably, for patients with breast cancer harbouring BRCA1/2 germline mutations, carboplatin proved superior to docetaxel (ORR: 68.0% vs 33.3%, *p* = 0.03; PFS: 6.8 vs 4.4 months, *p* = 0.002) (Tutt et al., 2018). The phase III CBCSG006 trial confirmed that the combination of cisplatin and gemcitabine demonstrated an improvement in ORR (64% vs 49%, *p* = 0.018) and median PFS (7.73 vs 6.47 months, *p* = 0.009) when compared to paclitaxel and gemcitabine in a cohort of 236 patients with metastatic TNBC, but did not improve median OS. Patients with gBRCA1/2 mutations showed notable enhancements in both ORR and PFS when treated with the platinum-gemcitabine combination, as illustrated by the results of a subgroup analysis (Hu et al., 2015). The mechanisms of action of eribulin and platinum drugs are different, and previous studies have not compared the effects of eribulin and platinum drugs as salvage therapy in recurrent metastatic triple-negative breast cancer, with or without BRCA1/2 gene mutations. In the study, we compared eribulin-based treatment with platinum-based treatment in unselected advanced TNBC patients. All populations displayed numerical advantages in ORR and DCR, including patients receiving both first-line and second-line and above treatment (Table 4). However, it is worth noting that following PSM, there were no statistically significant differences observed in ORR and DCR between eribulin-based treatment and platinum-based treatment (Table 5). This may be due to the study’s retrospective nature and the small sample size. After PSM, patients who received eribulin achieved a significantly longer median PFS and median OS compared to individuals who received platinum-based treatment (Figures 2G, H). In addition, in the multivariate analysis, the type of treatment (eribulin vs platinum, HR = 0.49, *p* = 0.039) and age <50 years (HR = 0.45, *p* = 0.016) were identified as independent predictors of PFS (Supplementary Figure S2). Considering eribulin’s unique mechanism of action, it has demonstrated a survival advantage over platinum chemotherapy in the entire population, without differentiating BRCA1/2 status. Our findings indicate that individuals under the age of 50 experience a greater benefit from eribulin-based treatment in terms of PFS. Additionally, eribulin exhibits milder side effects, such as nausea and vomiting, in contrast to platinum chemotherapy. However, our investigation involved a relatively small number of patients who had undergone BRCA1/2 testing, so the relationship between BRCA1/2 mutations and the efficacy of eribulin-based chemotherapy requires further investigation.

Combination therapy utilizing nab-paclitaxel has been researched as a first- and second-line chemotherapy option for patients with advanced TNBC. Findings from the tnAcity study showed that when compared to either nab-paclitaxel in combination with gemcitabine or gemcitabine in combination with carboplatin, nab-paclitaxel combined with carboplatin significantly extended PFS in patients with advanced TNBC (8.3 vs 5.5 months, *p* = 0.02 and 8.3 vs 6.0 months, *p* = 0.02) (Yardley et al., 2018). The GAP study compared nab-paclitaxel combined with cisplatin (AP, n = 127) and gemcitabine combined with cisplatin (GP, n = 126) as first-line treatment for metastatic TNBC. The median PFS was longer in the AP group than in the GP group (9.9 vs 7.5 months, HR = 0.66, *p* = 0.004). The AP group also demonstrated a statistically significant increase in ORR compared to the GP group (81.1% vs 55.9%, *p* < 0.001). Additionally, there was a tendency towards improvement in median OS in the AP group when compared to the GP group (26.3 vs 22.9 months, HR = 0.78, *p* = 0.21) (Wang et al., 2022). However, although nab-paclitaxel has shown some promise as salvage therapy in advanced TNBC (O'Shaughnessy et al., 2013), it remains unclear which is the optimal treatment option between eribulin and nab-paclitaxel. Although eribulin and nab-paclitaxel are both microtubule inhibitors, their mechanisms differ, and there may be differences in their efficacy for tumour suppression. However, previous studies have not compared the efficacy of eribulin and nab-paclitaxel for salvage therapy in recurrent metastatic triple-negative breast cancer. Our study showed that in both pre- and post-PSM populations (comprising total, first-line, and second-line and above populations), the ORR and DCR of eribulin treatment were slightly higher in comparison to nab-paclitaxel treatment (except for eribulin-based treatments having similar ORRs to nab-paclitaxel-based treatments in the second-line and above populations after PSM). However, there was no statistically significant difference observed, as in the other previous subgroups for similar reasons (Tables 4 and 5). After PSM, a considerable improvement in the median PFS and median OS was observed (Figures 2C, D). Multivariate analysis showed that the treatment type (eribulin vs nab-paclitaxel) and the combination with apatinib were independent predictors of improved PFS. In addition, the treatment type (eribulin vs nab-paclitaxel) was an autonomous predictor for OS (Supplementary Figure S1). Evidence suggests that eribulin is superior to nab-paclitaxel in terms of PFS and OS.In clinical practice, it is generally thought that nab-paclitaxel is appropriate as salvage therapy for advanced TNBC if paclitaxel (excluding nab-paclitaxel) was administered during the perioperative period and the DFI is less than12 months, paclitaxel (including nab-paclitaxel) was administered during the perioperative period and DFI is greater than or equal to12 months, or if paclitaxel (excluding nab-paclitaxel) was administered for salvage therapy. As our study comprised a limited sample of patients in the nab-paclitaxel cohort who met the above criteria, we did not compare the results of this group of patients with those treated with eribulin to reduce study bias. However, It is speculated that there may be cross-resistance between TNBC patients previously treated with taxanes and those treated with nab-paclitaxel. Therefore, eribulin, which affects microtubules but has a different mechanism to taxanes, may provide a better survival benefit. Further studies with larger sample sizes are needed to validate this hypothesis.

In our study, the overall safety profile of eribulin was favourable, with no toxic deaths or treatment discontinuations. The most prevalent haematological AEs associated with eribulin were neutropenia (81.0%), anaemia (35.7%), and thrombocytopenia (16.7%). Amongst the non-hematological toxicities, the most frequent was alanine aminotransferase (ALT) elevation in 38.1%, followed by fatigue in 35.7%, alopecia in 33.3%, hand-foot syndrome in 23.8%, peripheral neuropathy in 11.9% and nausea and vomiting in 7.1%.Grade 3/4 haematological toxicities were predominantly neutropenia (26.2%), with infrequent cases of thrombocytopenia (2.4%). The non-haematological toxicities observed at Grade 3/4 were elevated ALT (7.1%) and fatigue (2.4%). No instances of grade 3/4 anaemia, peripheral neuropathy, nausea/vomiting, diarrhoea, or hand-foot syndrome were observed. Compared to other chemotherapy regimens featuring taxanes or platinum, the eribulin-based group exhibited significantly lower rates of any-grade anaemia, peripheral neuropathy, nausea or vomiting, and alopecia (Supplementary Tables S1, S2). Research indicates that dose adjustments of eribulin could be taken into consideration for recurring or severe grade 3/4 haematological adverse events that cause hindrances in subsequent treatment. Mostly, non-haematological adverse events were observed to be mild to moderate in intensity and can be managed with supportive therapy and/or dose adjustment.

## 5 Conclusion

Our study suggested that Eribulin-based treatments yielded a promising response rate and resulted in substantial improvement in PFS and OS when compared to other chemotherapy regimens, including platinum and nab-paclitaxel, for patients with advanced TNBC. It may be worthwhile to consider adjusting the dose of eribulin in cases of recurrent or severe grade 3/4 haematological AEs that impede subsequent treatment. The majority of non-haematological adverse events are of mild to moderate severity and can be managed through supportive measures or dosage adjustments. To sum up, eribulin was well tolerated. Nevertheless, due to the limited number of cases included and the extended follow-up time in this study, there may be some bias and further investigation through large prospective studies is needed.

## Data Availability

The original contributions presented in the study are included in the article/Supplementary Material, further inquiries can be directed to the corresponding author.
