# Supplementary material for: Feasibility and tolerability of eribulin-based chemotherapy versus other chemotherapy regimens for patients with metastatic triple-negative breast cancer: a single-centre retrospective study
Source: Front Cell Dev Biol. 2024 Feb 22;12:1313610. doi: 10.3389/fcell.2024.1313610 (PMC10936577; doi:10.3389/fcell.2024.1313610)
Supplement: Supplementary file 1 [file DataSheet1.ZIP › R. code/2. Kaplan-Meier Analysis/KM Cohort 1-Eribulin vs ABX/Figure-EA.pptx]

## Slide 1
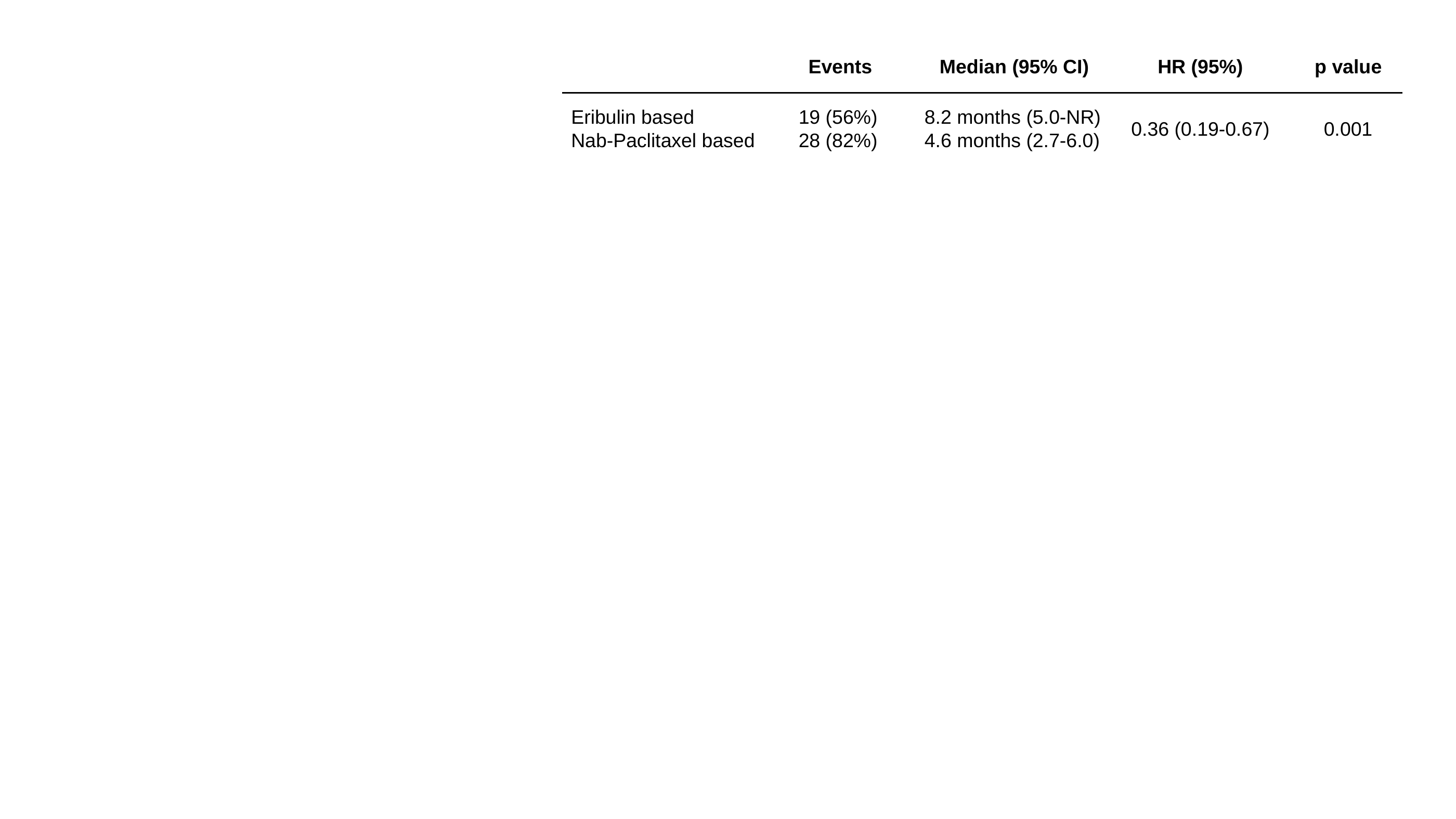

Events
Median (95% CI)
HR (95%)
p value
Eribulin based
Nab-Paclitaxel based
8.2 months (5.0-NR)
4.6 months (2.7-6.0)
19 (56%)
28 (82%)
0.36 (0.19-0.67)
0.001

## Slide 2
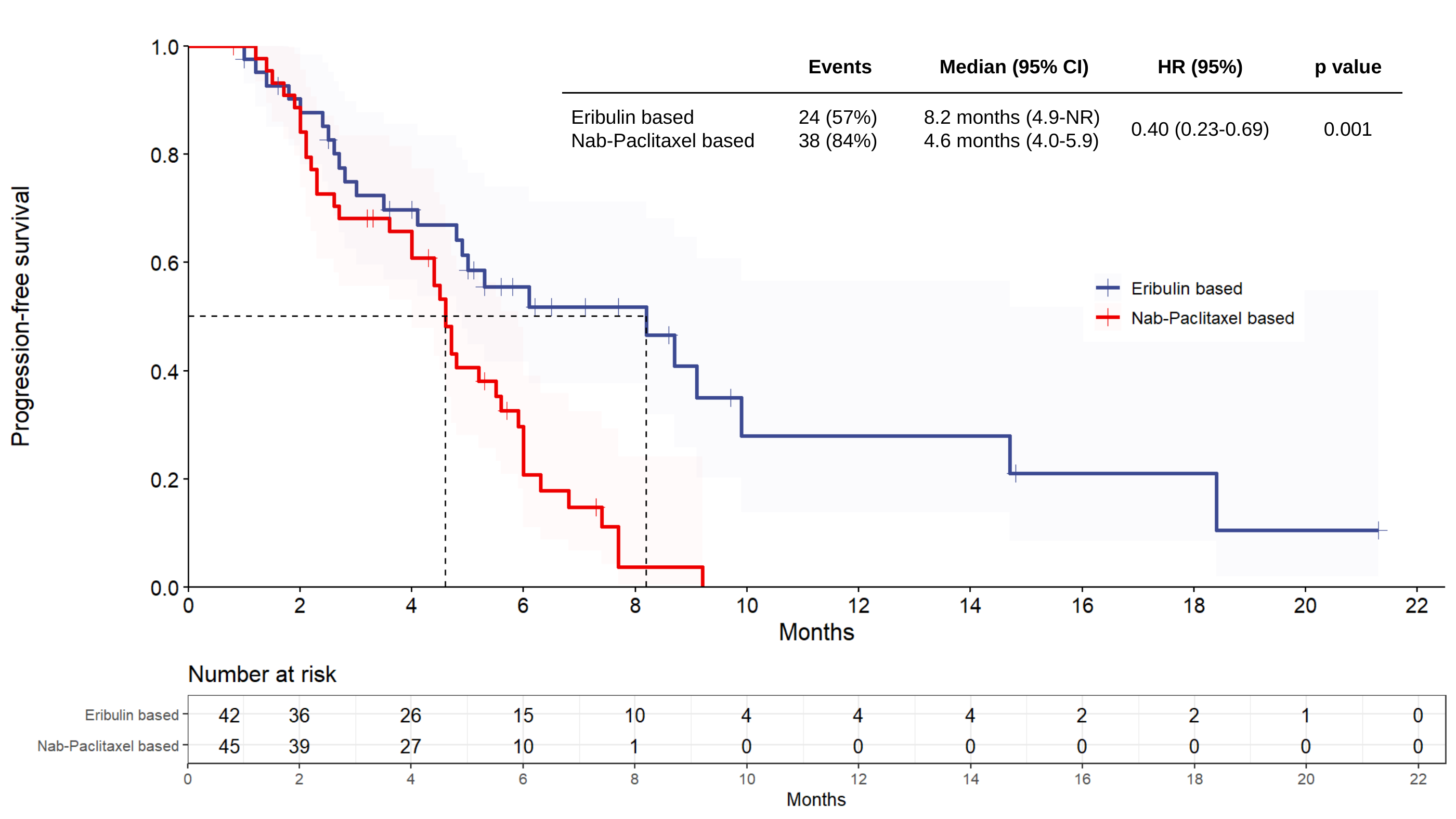

Events
Median (95% CI)
HR (95%)
p value
Eribulin based
Nab-Paclitaxel based
8.2 months (4.9-NR)
4.6 months (4.0-5.9)
24 (57%)
38 (84%)
0.40 (0.23-0.69)
0.001

## Slide 3
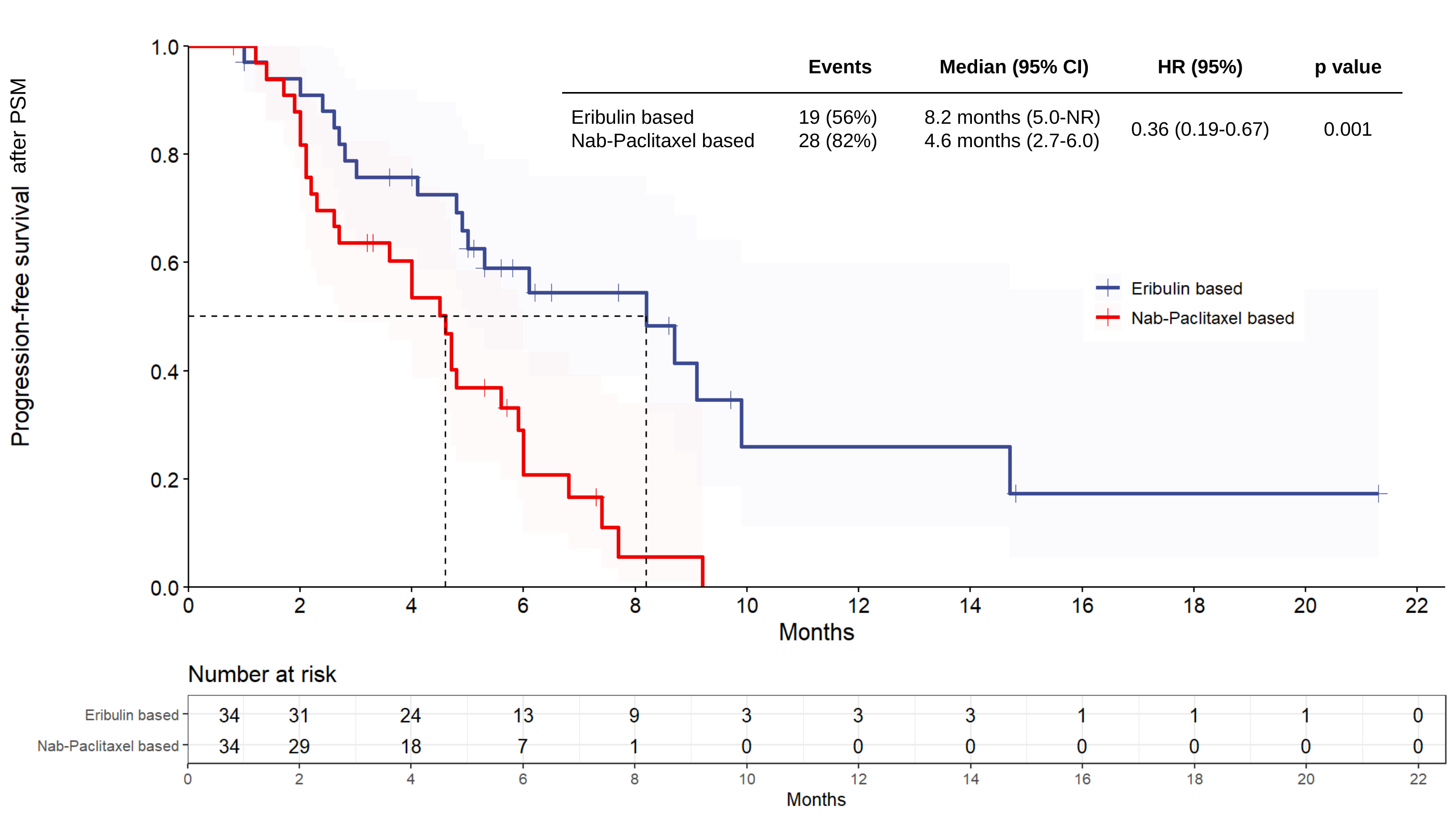

Events
Median (95% CI)
HR (95%)
p value
Eribulin based
Nab-Paclitaxel based
8.2 months (5.0-NR)
4.6 months (2.7-6.0)
19 (56%)
28 (82%)
0.36 (0.19-0.67)
0.001
after PSM

## Slide 4
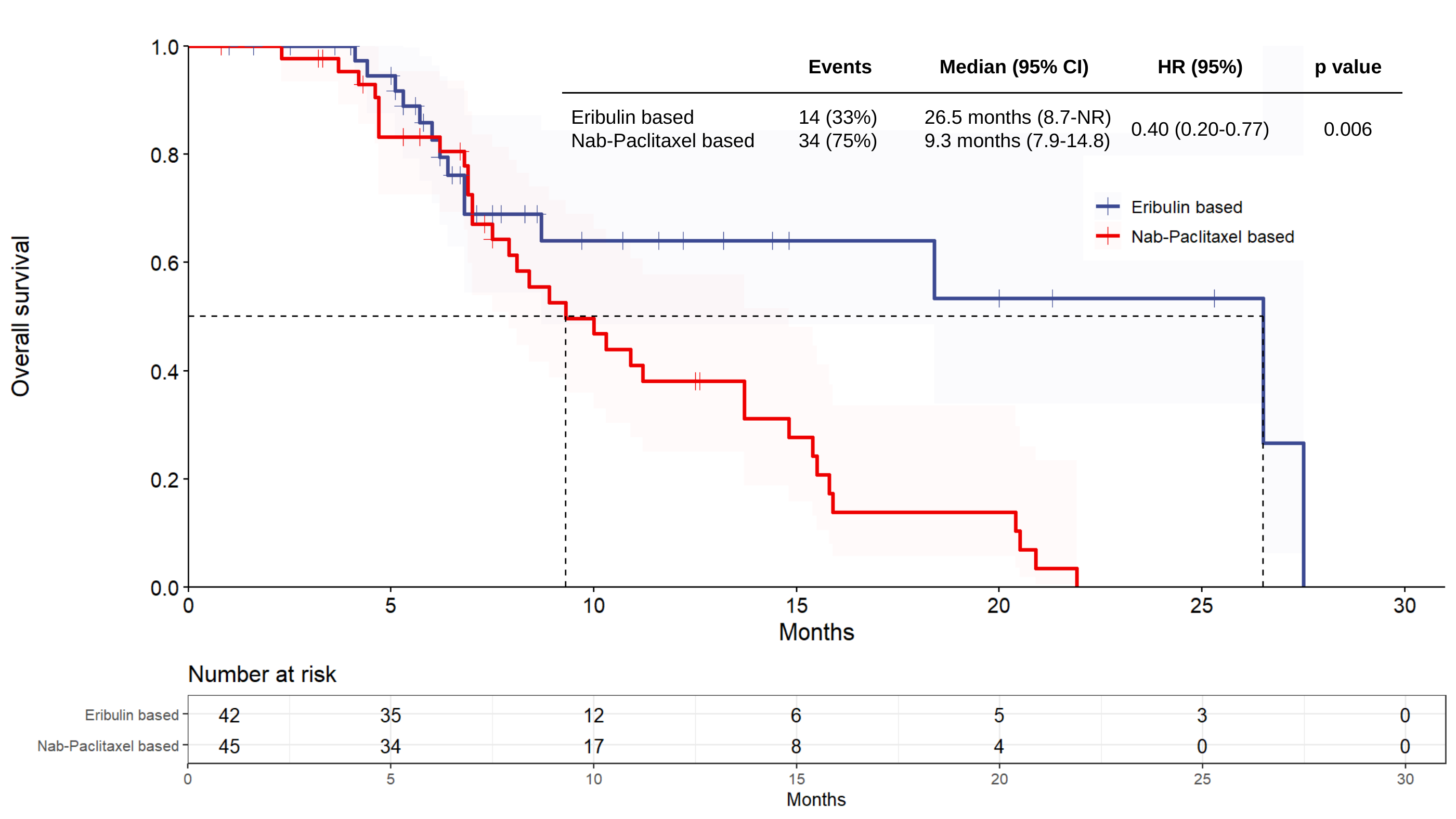

Events
Median (95% CI)
HR (95%)
p value
Eribulin based
Nab-Paclitaxel based
26.5 months (8.7-NR)
9.3 months (7.9-14.8)
14 (33%)
34 (75%)
0.40 (0.20-0.77)
0.006

## Slide 5
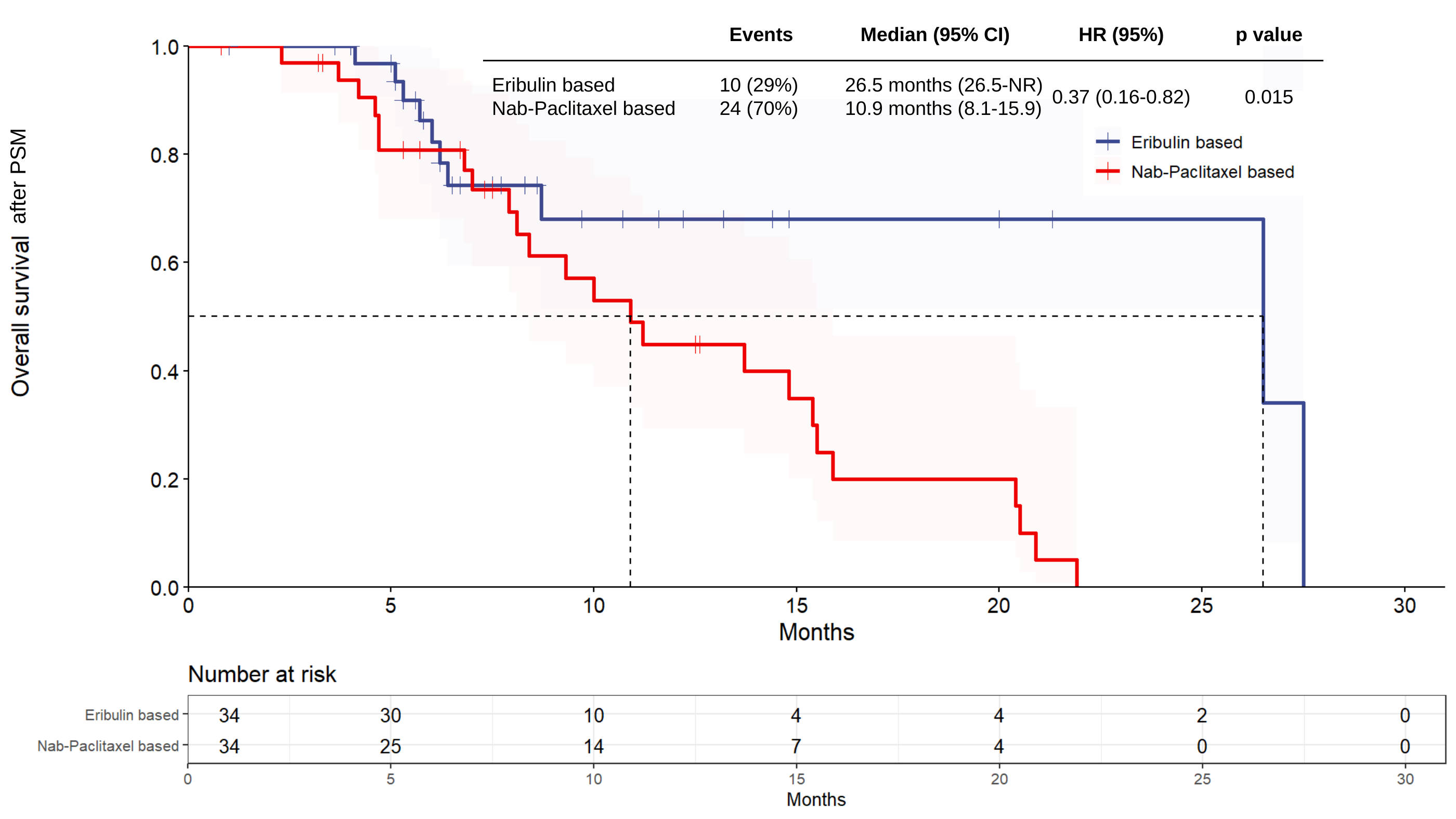

Events
Median (95% CI)
HR (95%)
p value
Eribulin based
Nab-Paclitaxel based
26.5 months (26.5-NR)
10.9 months (8.1-15.9)
10 (29%)
24 (70%)
0.37 (0.16-0.82)
0.015
after PSM
