# Supplementary material for: Feasibility and tolerability of eribulin-based chemotherapy versus other chemotherapy regimens for patients with metastatic triple-negative breast cancer: a single-centre retrospective study
Source: Front Cell Dev Biol. 2024 Feb 22;12:1313610. doi: 10.3389/fcell.2024.1313610 (PMC10936577; doi:10.3389/fcell.2024.1313610)
Supplement: Supplementary file 1 [file DataSheet1.ZIP › R. code/2. Kaplan-Meier Analysis/KM Cohort 2-Eribulin vs Platinum/Figure-EP.pptx]

## Slide 1
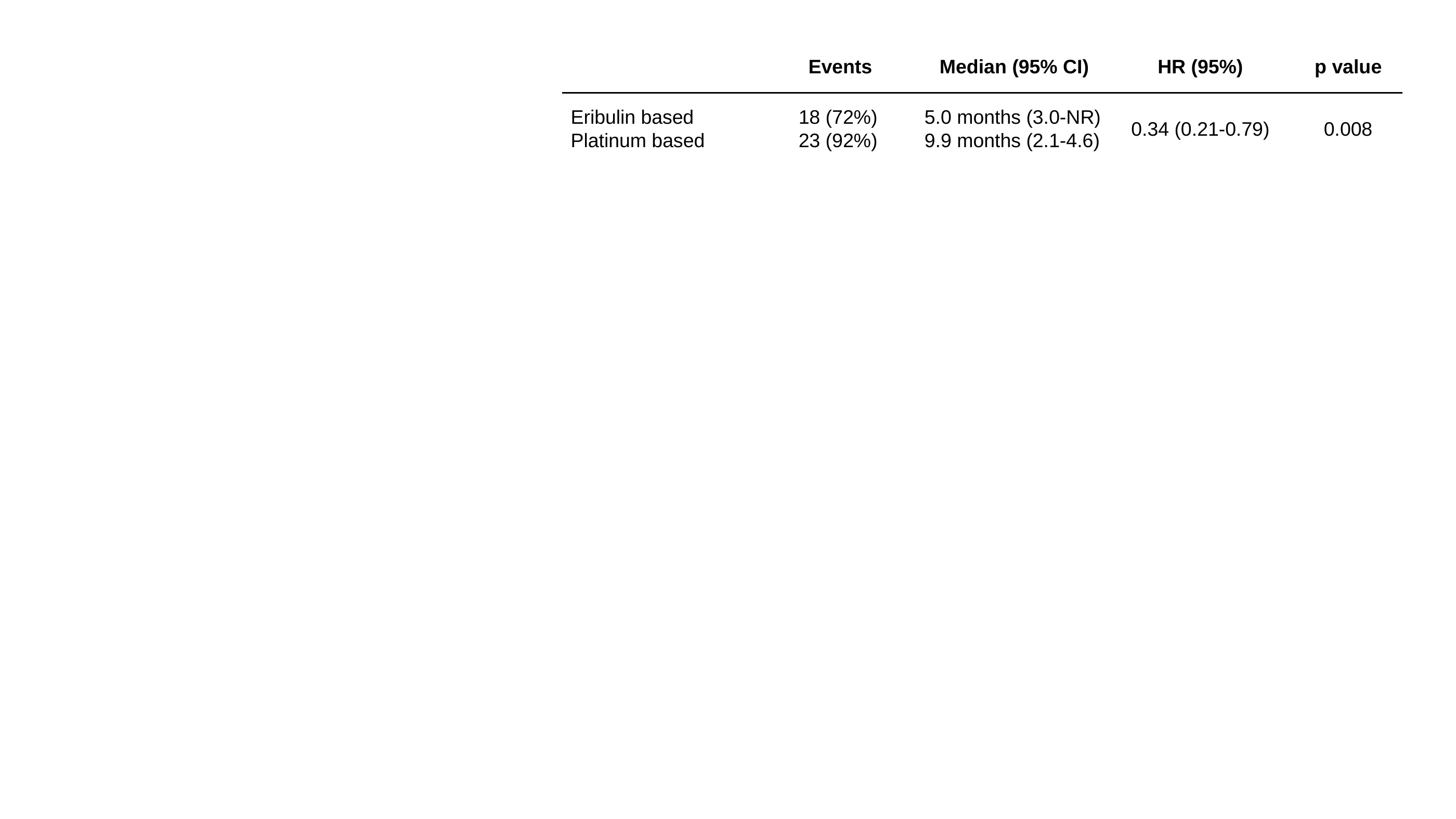

Events
Median (95% CI)
HR (95%)
p value
Eribulin based
Platinum based
5.0 months (3.0-NR)
9.9 months (2.1-4.6)
18 (72%)
23 (92%)
0.34 (0.21-0.79)
0.008

## Slide 2
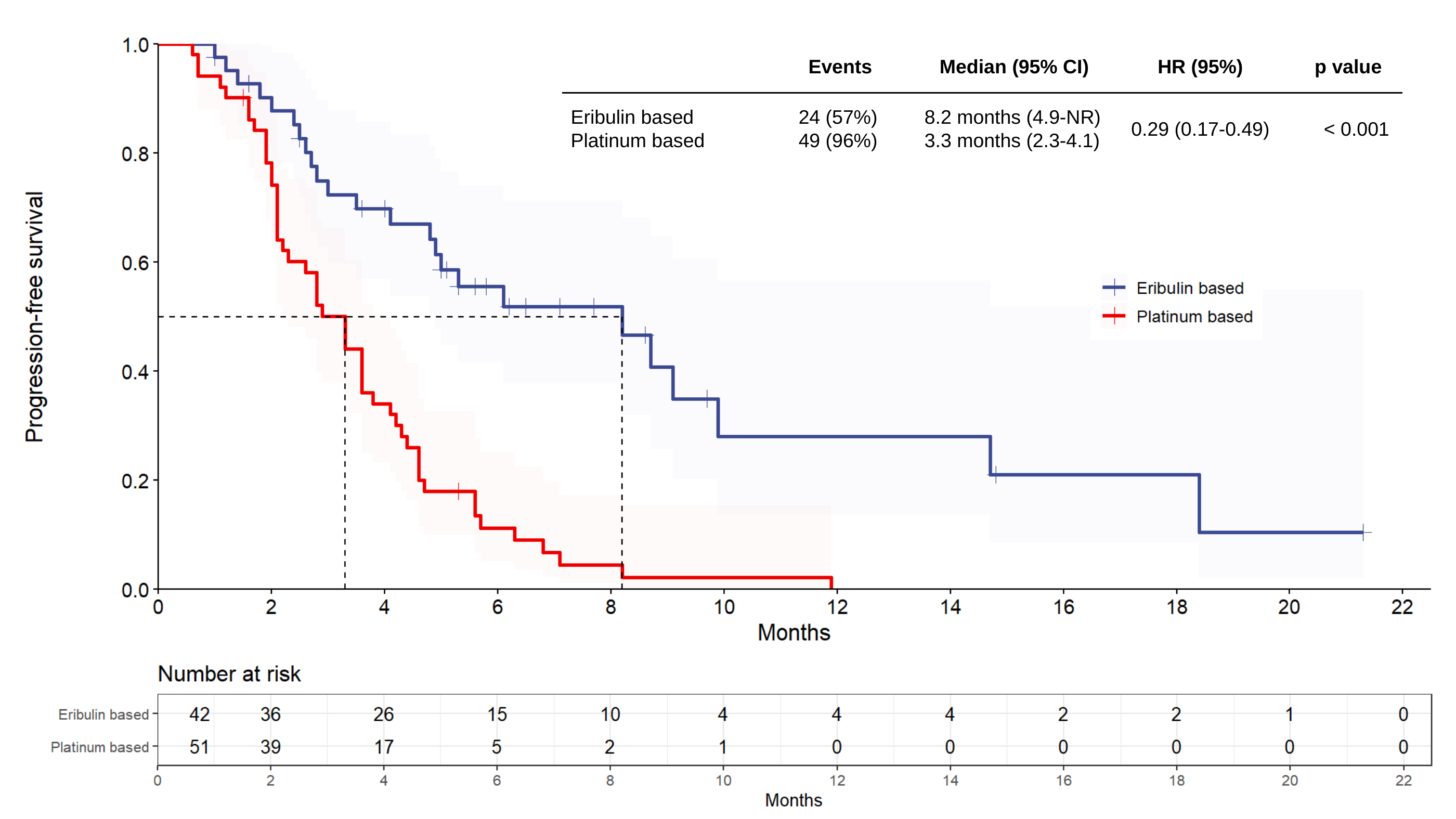

Events
Median (95% CI)
HR (95%)
p value
Eribulin based
Platinum based
8.2 months (4.9-NR)
3.3 months (2.3-4.1)
24 (57%)
49 (96%)
0.29 (0.17-0.49)
< 0.001

## Slide 3
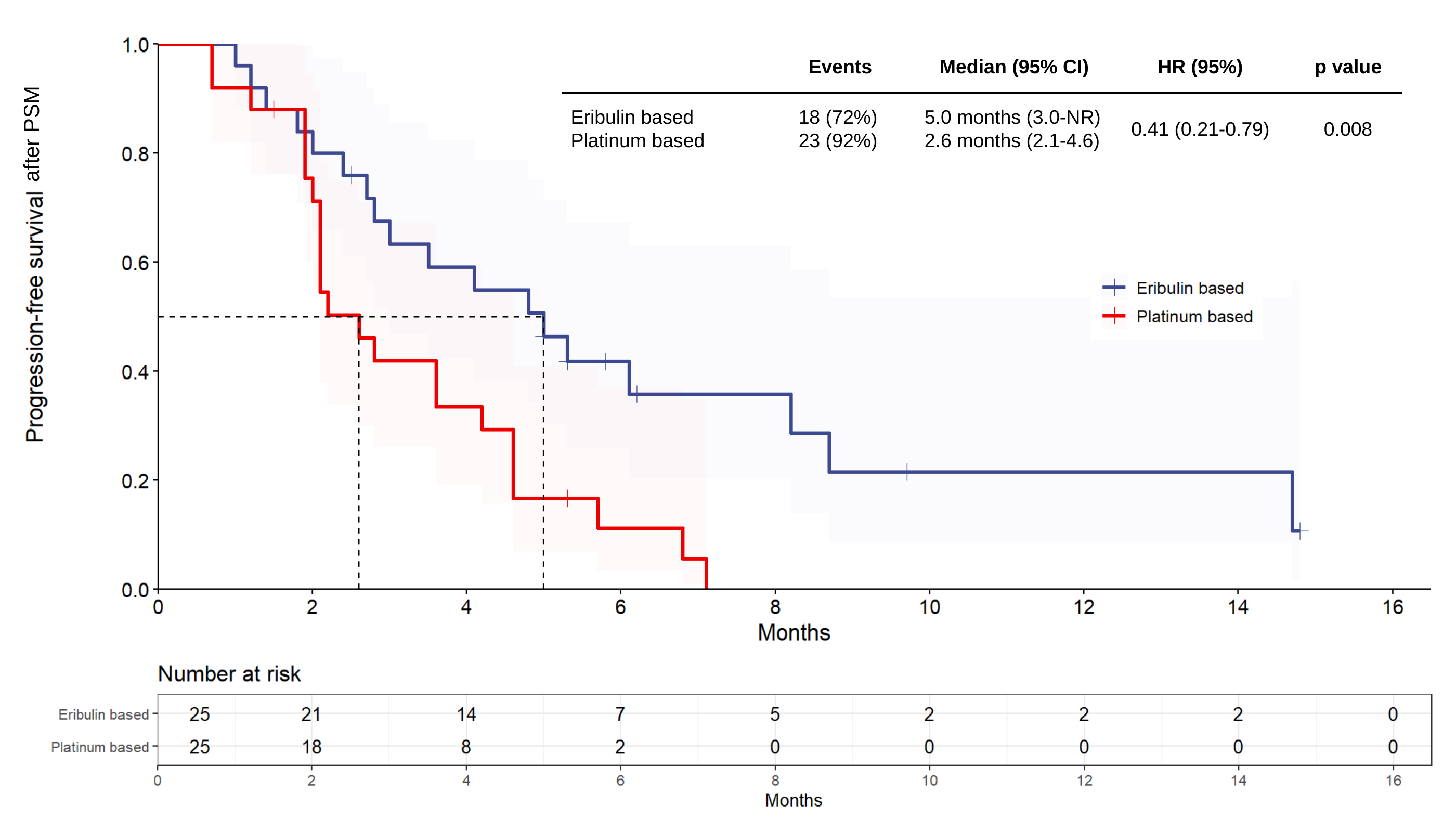

Events
Median (95% CI)
HR (95%)
p value
Eribulin based
Platinum based
5.0 months (3.0-NR)
2.6 months (2.1-4.6)
18 (72%)
23 (92%)
0.41 (0.21-0.79)
0.008
after PSM

## Slide 4
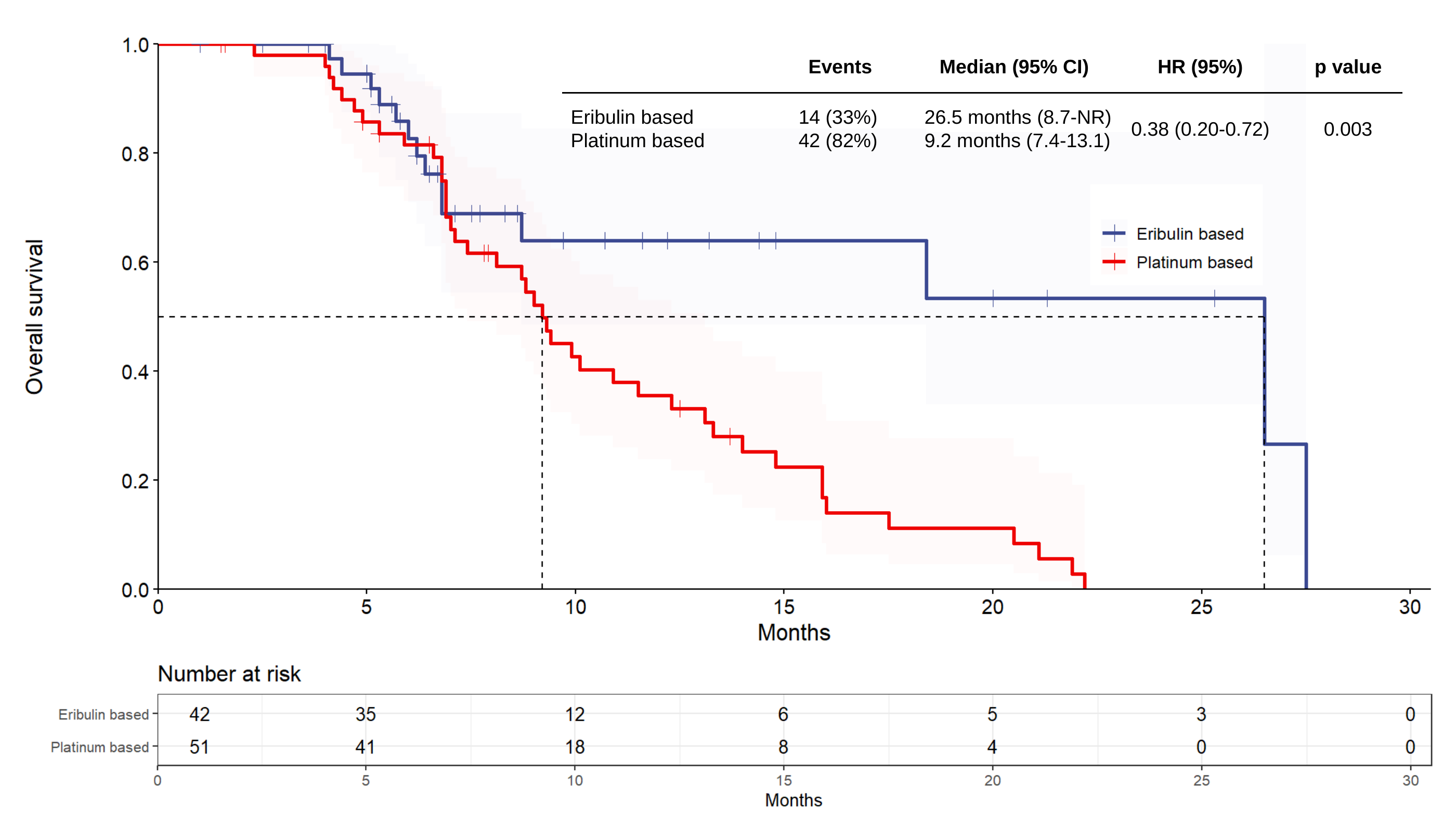

Events
Median (95% CI)
HR (95%)
p value
Eribulin based
Platinum based
26.5 months (8.7-NR)
9.2 months (7.4-13.1)
14 (33%)
42 (82%)
0.38 (0.20-0.72)
0.003

## Slide 5
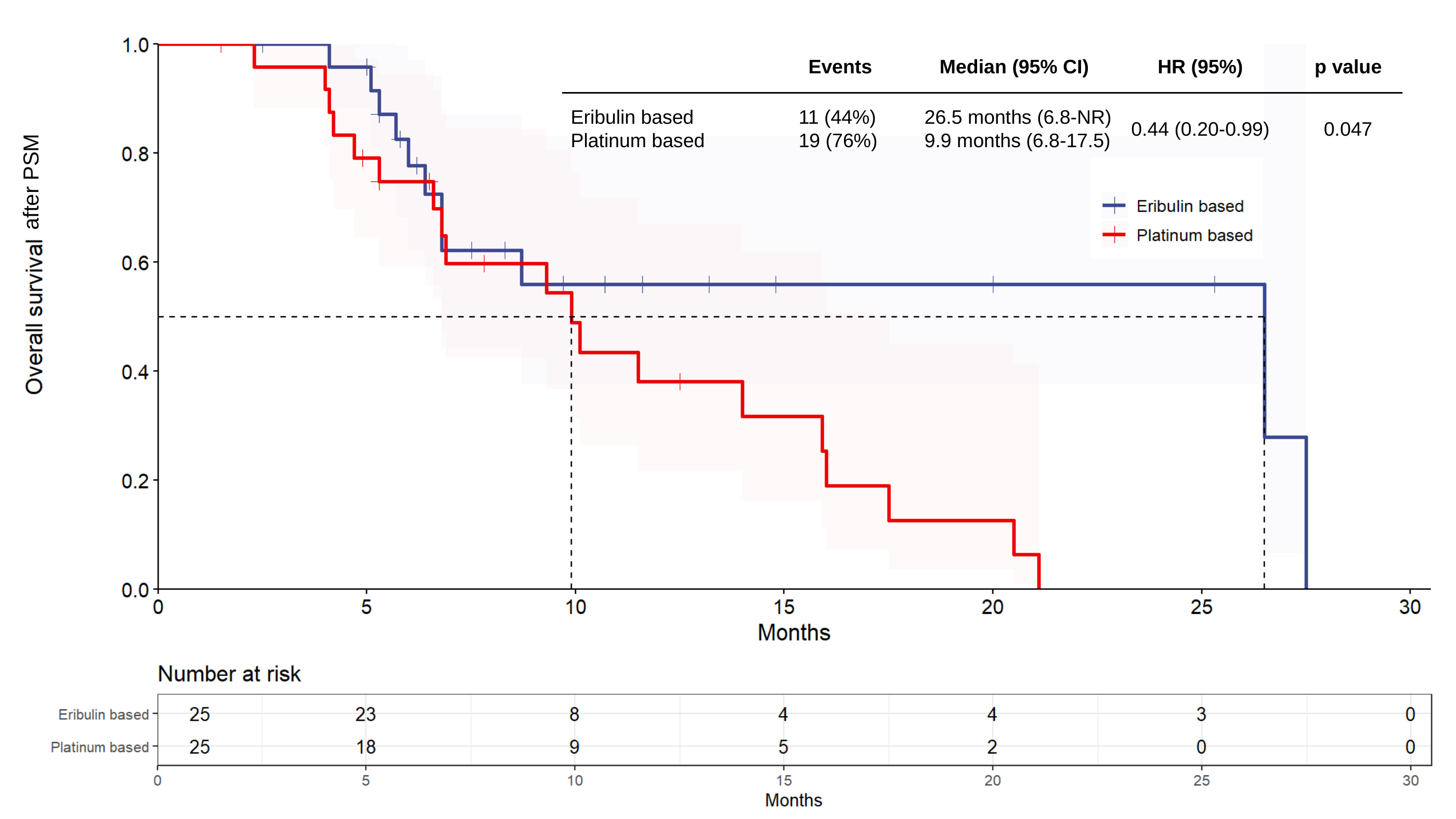

Events
Median (95% CI)
HR (95%)
p value
Eribulin based
Platinum based
26.5 months (6.8-NR)
9.9 months (6.8-17.5)
11 (44%)
19 (76%)
0.44 (0.20-0.99)
0.047
after PSM
