# Supplementary material for: Feasibility and tolerability of eribulin-based chemotherapy versus other chemotherapy regimens for patients with metastatic triple-negative breast cancer: a single-centre retrospective study
Source: Front Cell Dev Biol. 2024 Feb 22;12:1313610. doi: 10.3389/fcell.2024.1313610 (PMC10936577; doi:10.3389/fcell.2024.1313610)
Supplement: Supplementary file 1 [file DataSheet1.ZIP › R. code/2. Kaplan-Meier Analysis/KM Cohort 3-Eribulin vs OtherChemotherapy/Figure-EO.pptx]

## Slide 1
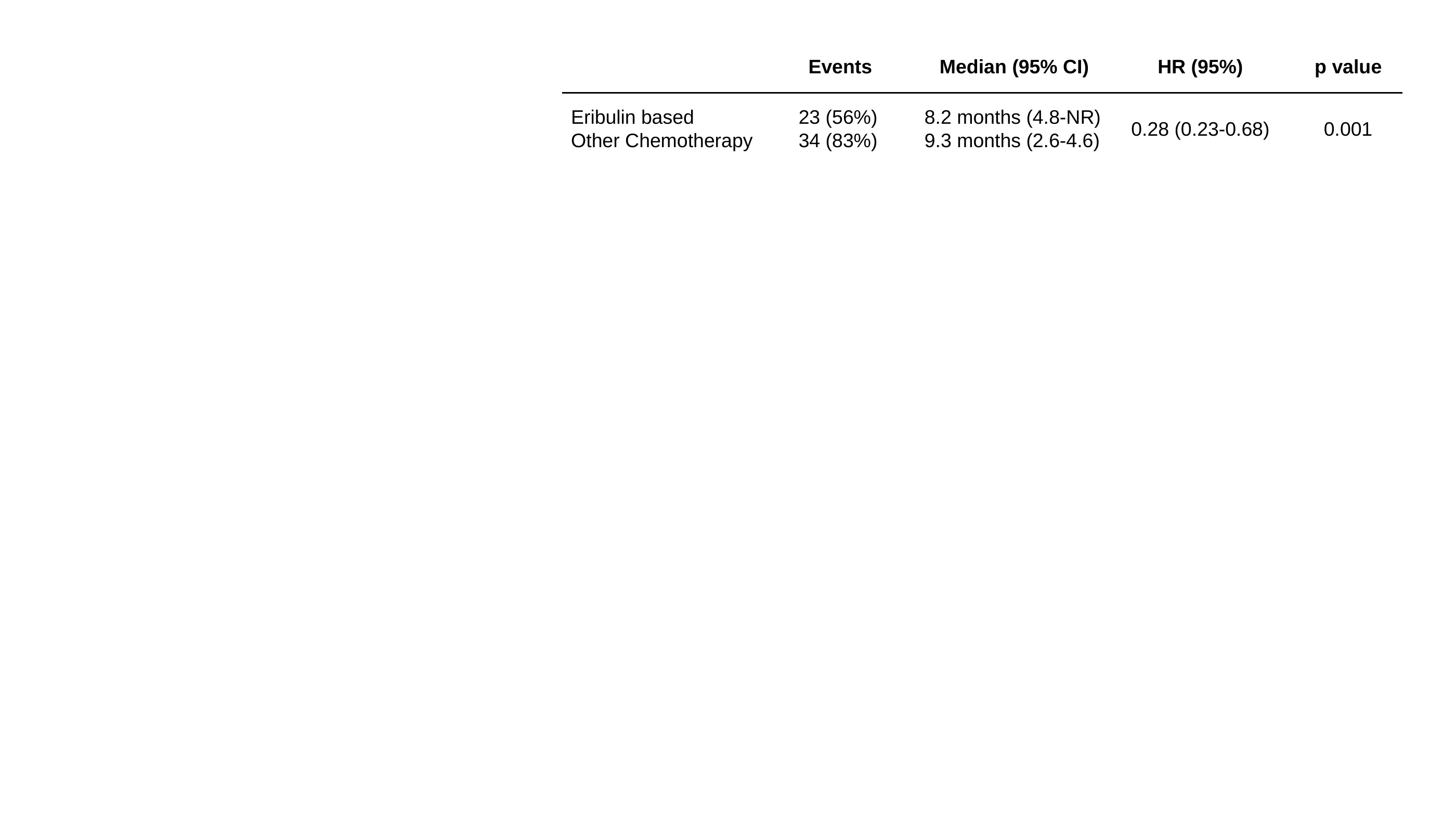

Events
Median (95% CI)
HR (95%)
p value
Eribulin based
Other Chemotherapy
8.2 months (4.8-NR)
9.3 months (2.6-4.6)
23 (56%)
34 (83%)
0.28 (0.23-0.68)
0.001

## Slide 2
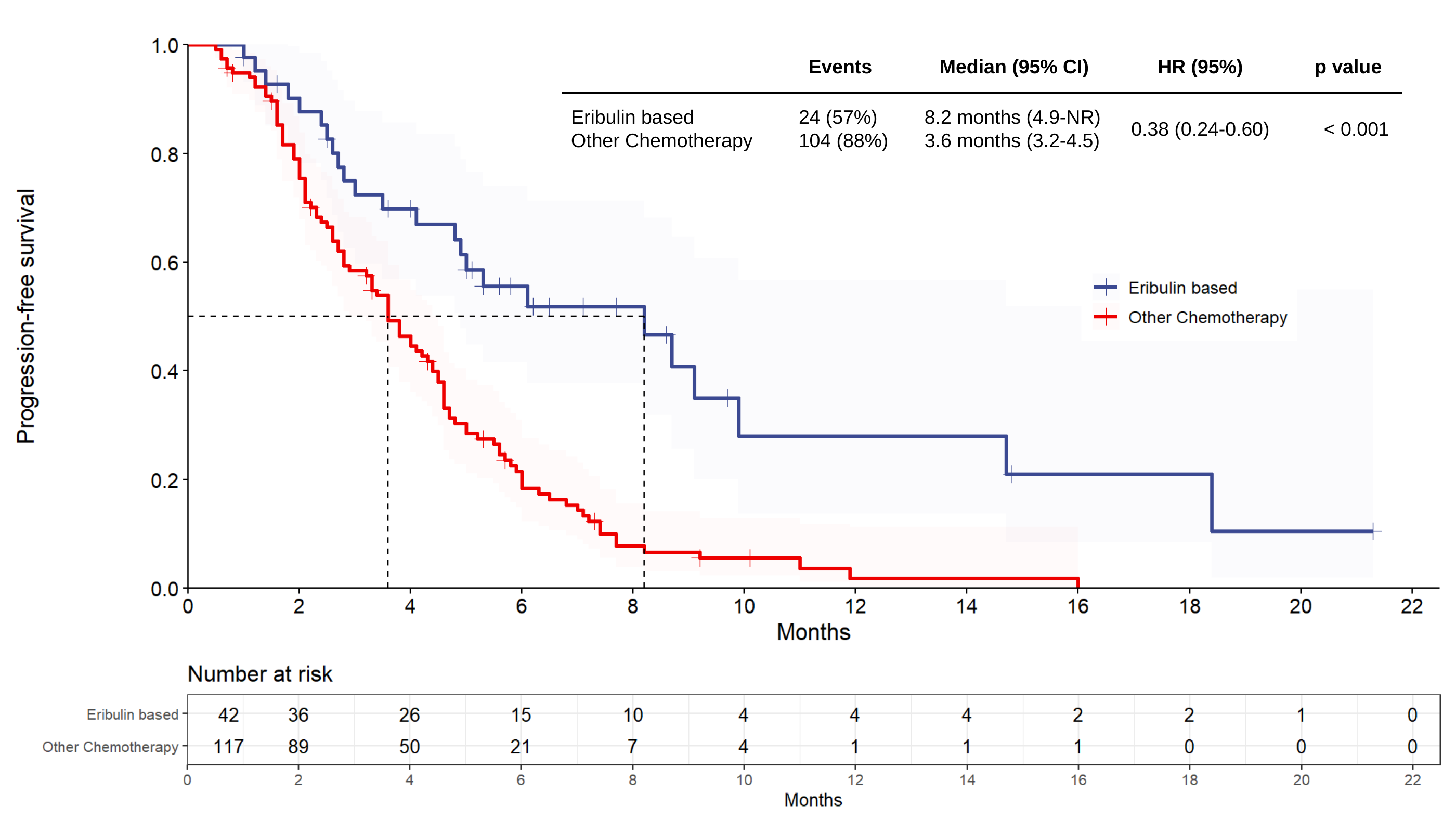

Events
Median (95% CI)
HR (95%)
p value
Eribulin based
Other Chemotherapy
8.2 months (4.9-NR)
3.6 months (3.2-4.5)
24 (57%)
104 (88%)
0.38 (0.24-0.60)
< 0.001

## Slide 3
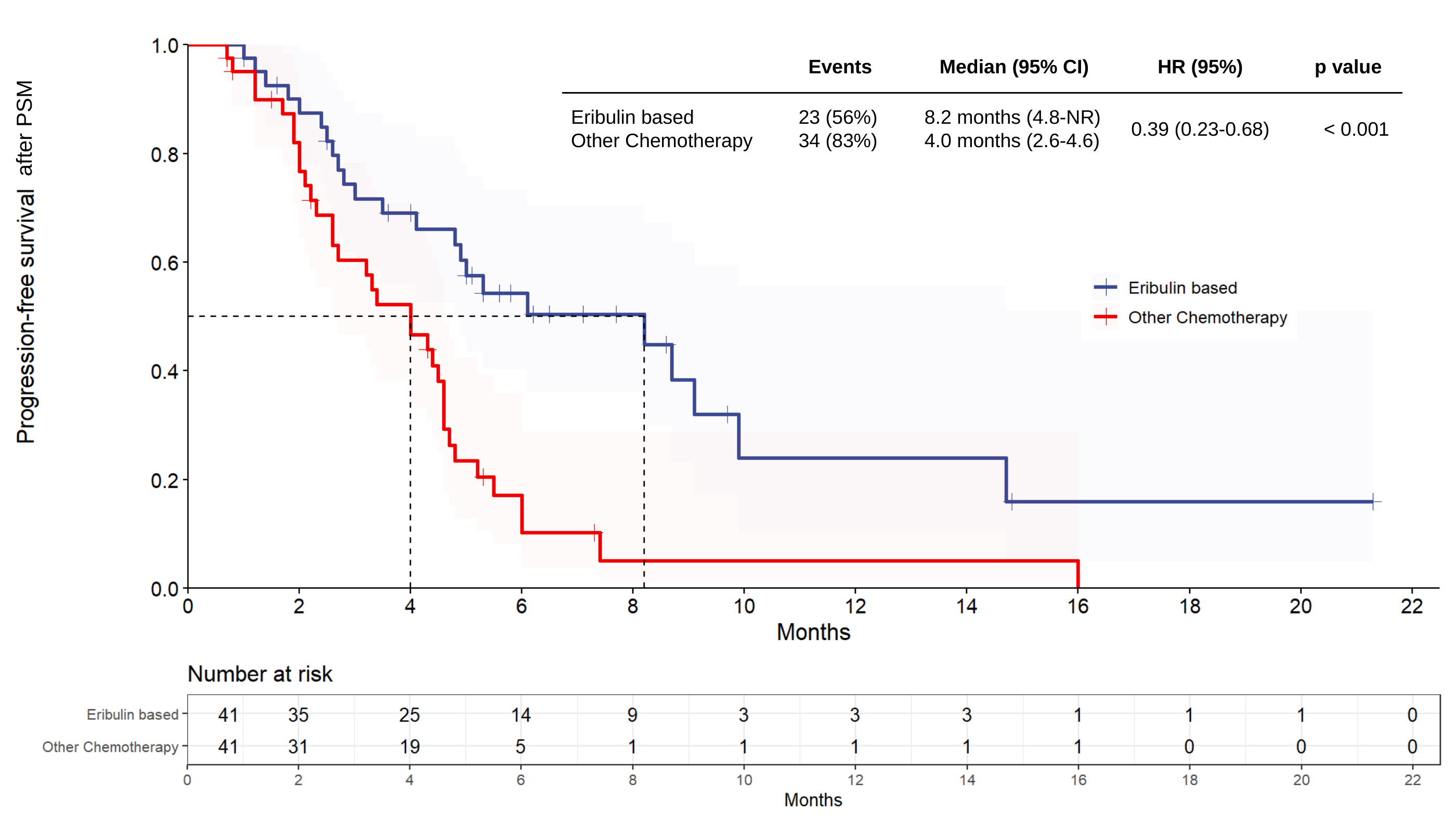

Events
Median (95% CI)
HR (95%)
p value
Eribulin based
Other Chemotherapy
8.2 months (4.8-NR)
4.0 months (2.6-4.6)
23 (56%)
34 (83%)
0.39 (0.23-0.68)
< 0.001
after PSM

## Slide 4
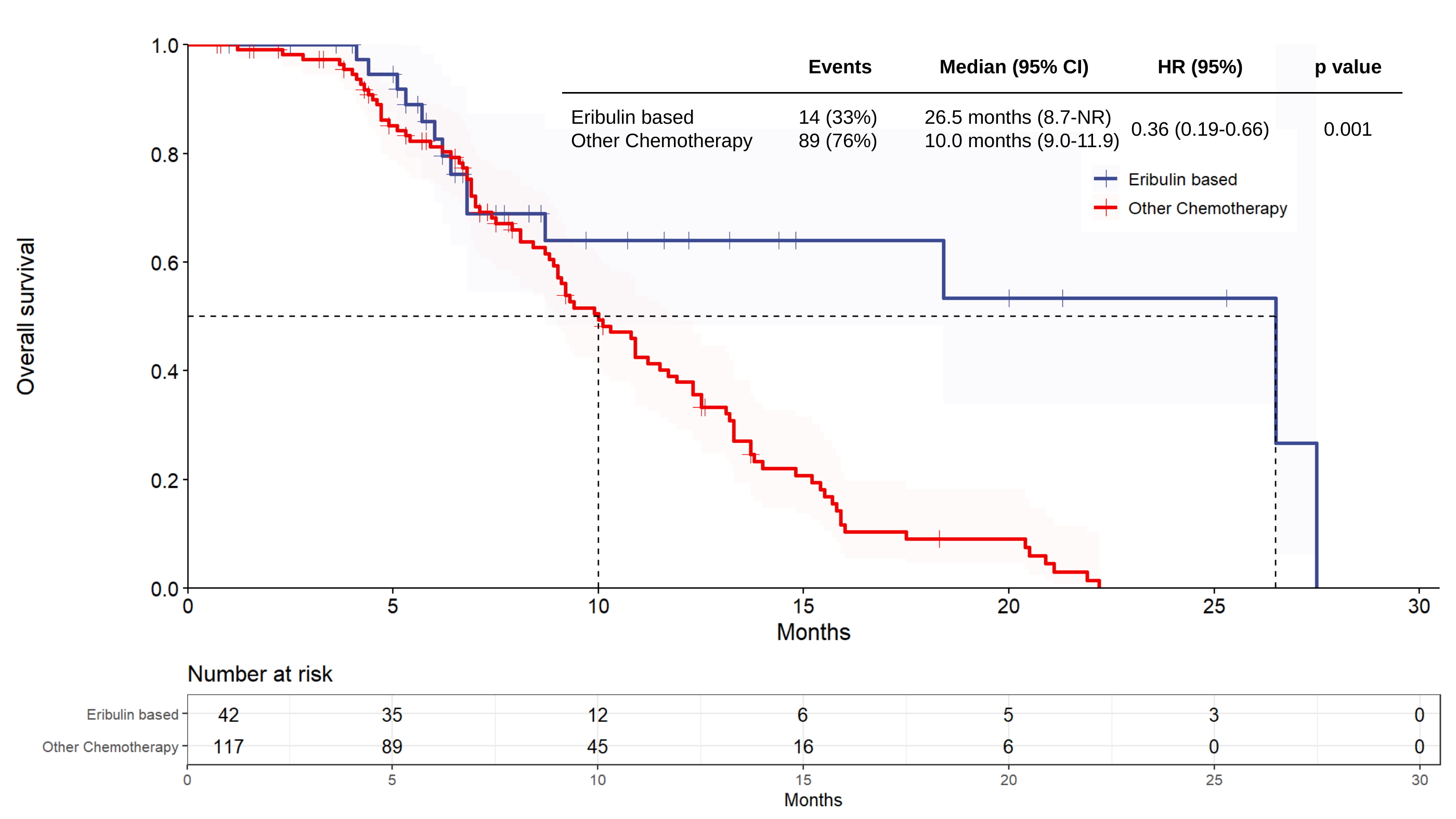

Events
Median (95% CI)
HR (95%)
p value
Eribulin based
Other Chemotherapy
26.5 months (8.7-NR)
10.0 months (9.0-11.9)
14 (33%)
89 (76%)
0.36 (0.19-0.66)
0.001

## Slide 5
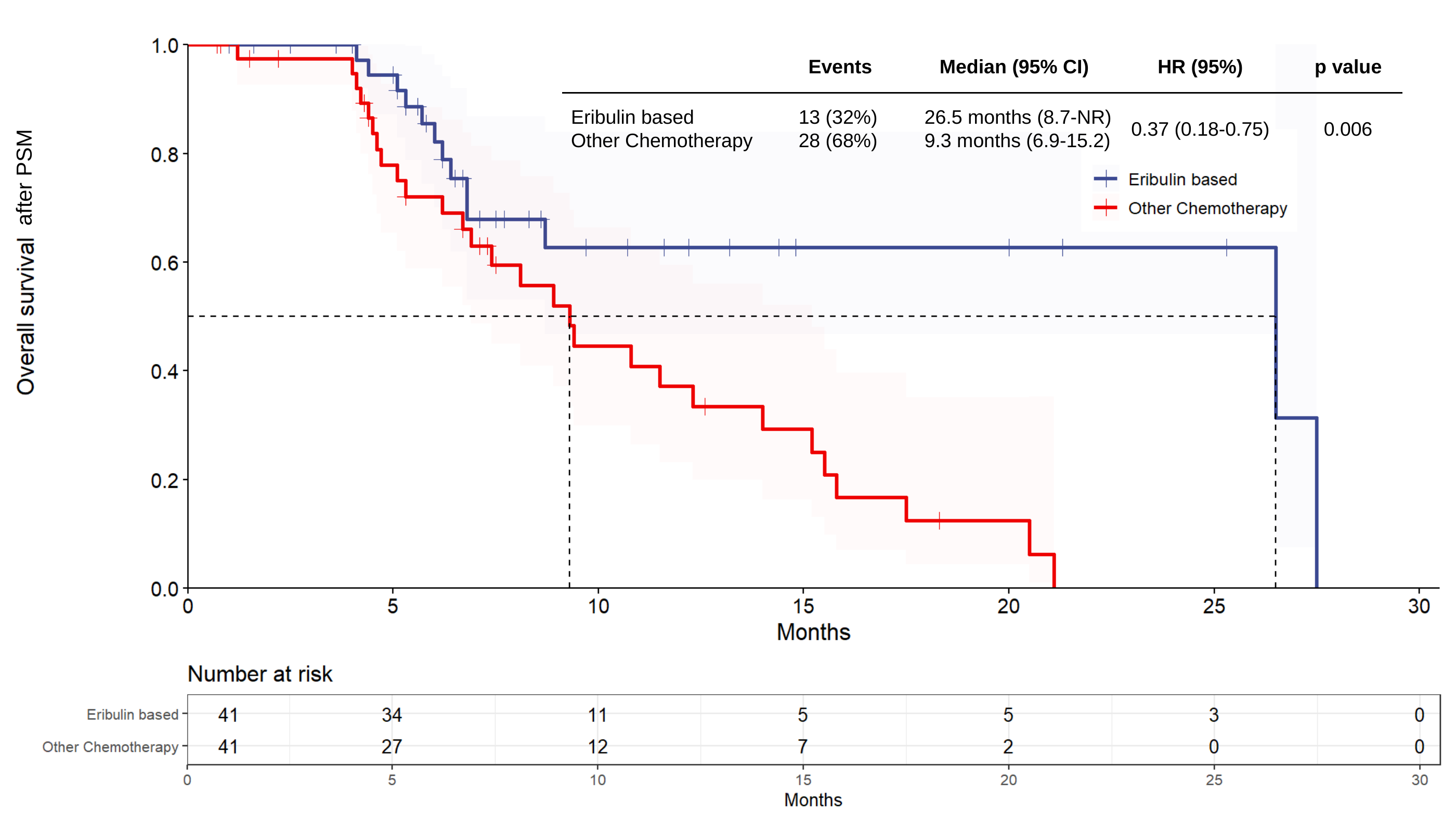

Events
Median (95% CI)
HR (95%)
p value
Eribulin based
Other Chemotherapy
26.5 months (8.7-NR)
9.3 months (6.9-15.2)
13 (32%)
28 (68%)
0.37 (0.18-0.75)
0.006
after PSM
