# Supplementary material for: Feasibility and tolerability of eribulin-based chemotherapy versus other chemotherapy regimens for patients with metastatic triple-negative breast cancer: a single-centre retrospective study
Source: Front Cell Dev Biol. 2024 Feb 22;12:1313610. doi: 10.3389/fcell.2024.1313610 (PMC10936577; doi:10.3389/fcell.2024.1313610)
Supplement: Supplementary file 2 [file DataSheet2.ZIP › source images/Figure 2 (ABCD). KM Analysis of PFS&OS for Eribulin and Nab-paclitaxel.pdf]

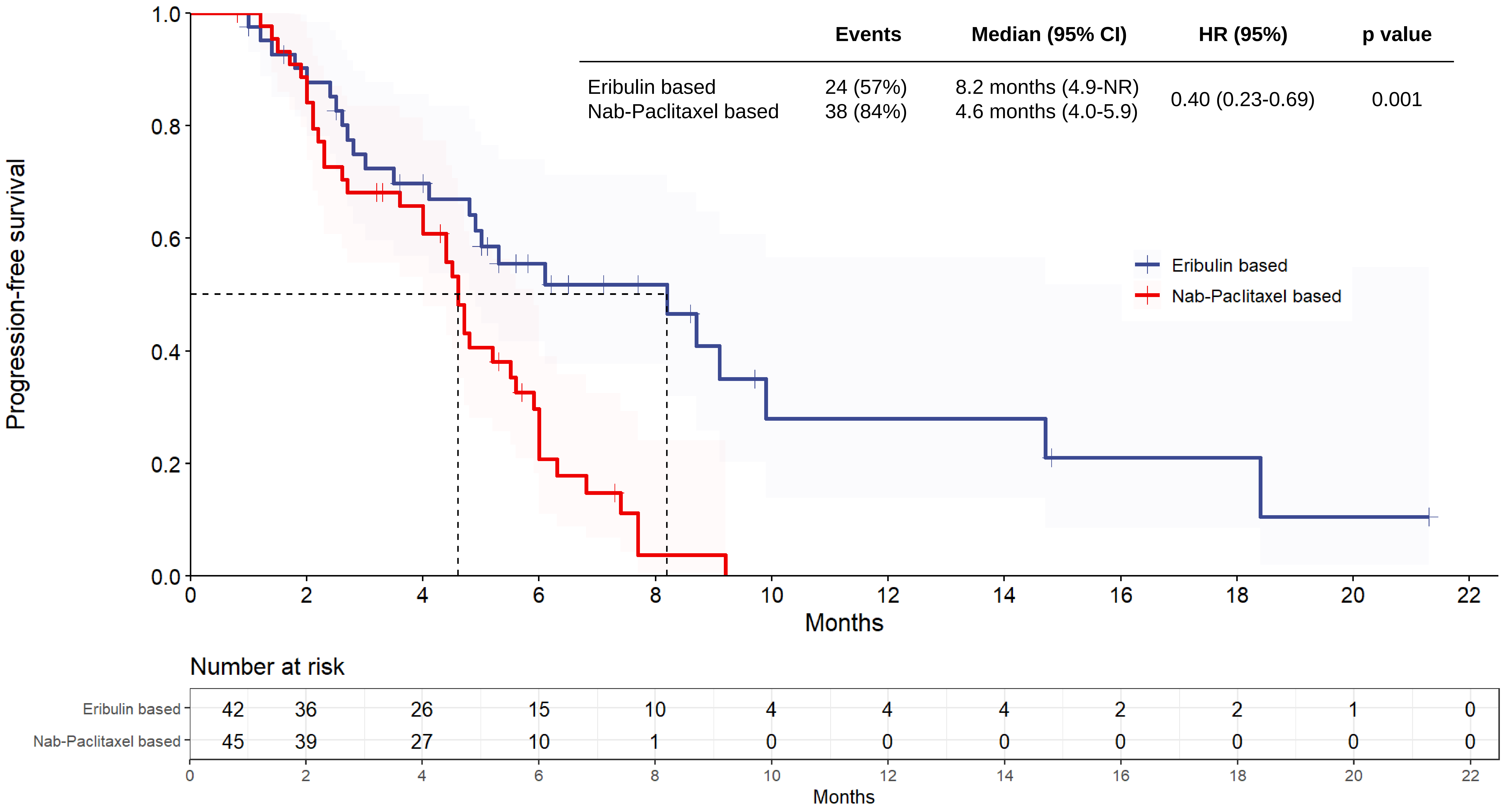

Progression-free survival
1.0
0.8
0.6
0.4
0.2
0.0
Events
Median (95% CI)
HR (95%)
p value
Eribulin based
24 (57%)
8.2 months (4.9-NR)
Nab-Paclitaxel based
38 (84%)
4.6 months (4.0-5.9)
0.40 (0.23-0.69)
0.001
Eribulin based
Nab-Paclitaxel based
0 2 4 6 8 10 12 14 16 18 20 22
Months
Number at risk
Eribulin based 42 36 26 15 10 4 4 4 2 2 1 0
Nab-Paclitaxel based 45 39 27 10 1 0 0 0 0 0 0 0
0 2 4 6 8 10 12 14 16 18 20 22
Months

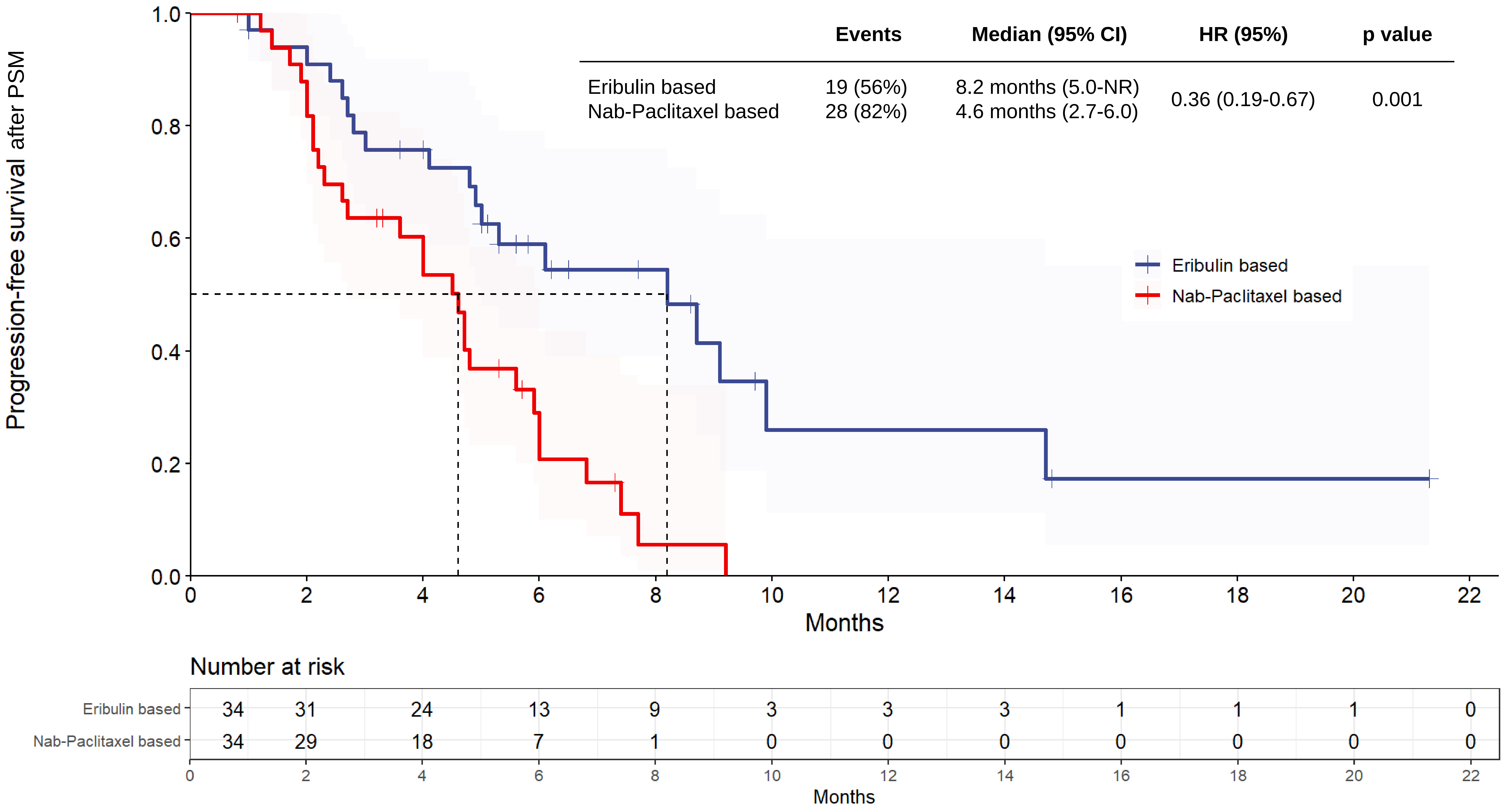

Progression-free survival after PSM
1.0
0.8
0.6
0.4
0.2
0.0
Events
Median (95% CI)
HR (95%)
p value
Eribulin based
19 (56%)
8.2 months (5.0-NR)
Nab-Paclitaxel based
28 (82%)
4.6 months (2.7-6.0)
0.36 (0.19-0.67)
0.001
Eribulin based
Nab-Paclitaxel based
0 2 4 6 8 10 12 14 16 18 20 22
Months
Number at risk
Eribulin based 34 31 24 13 9 3 3 3 1 1 1 0
Nab-Paclitaxel based 34 29 18 7 1 0 0 0 0 0 0 0
0 2 4 6 8 10 12 14 16 18 20 22
Months

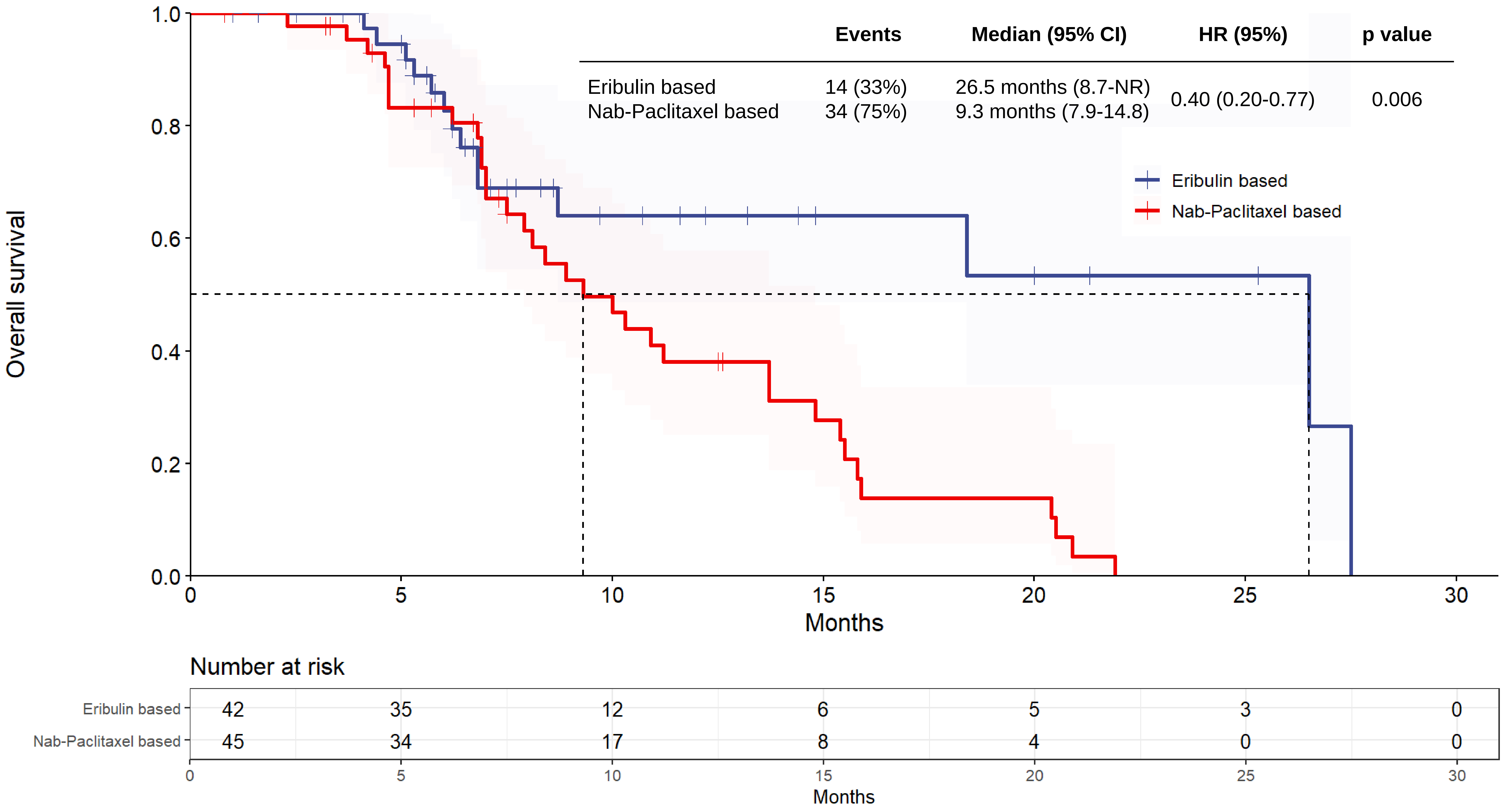
Events
Median (95% CI)
HR (95%)
p value
Eribulin based
14 (33%)
26.5 months (8.7-NR)
Nab-Paclitaxel based
34 (75%)
9.3 months (7.9-14.8)
0.40 (0.20-0.77)
0.006
Eribulin based
Nab-Paclitaxel based
Overall survival
1.0
0.8
0.6
0.4
0.2
0.0
0
5
10
15
20
25
30
Months
Number at risk
Eribulin based 42 35 12 6 5 3 0
Nab-Paclitaxel based 45 34 17 8 4 0 0
0
5
10
15
20
25
30
Months

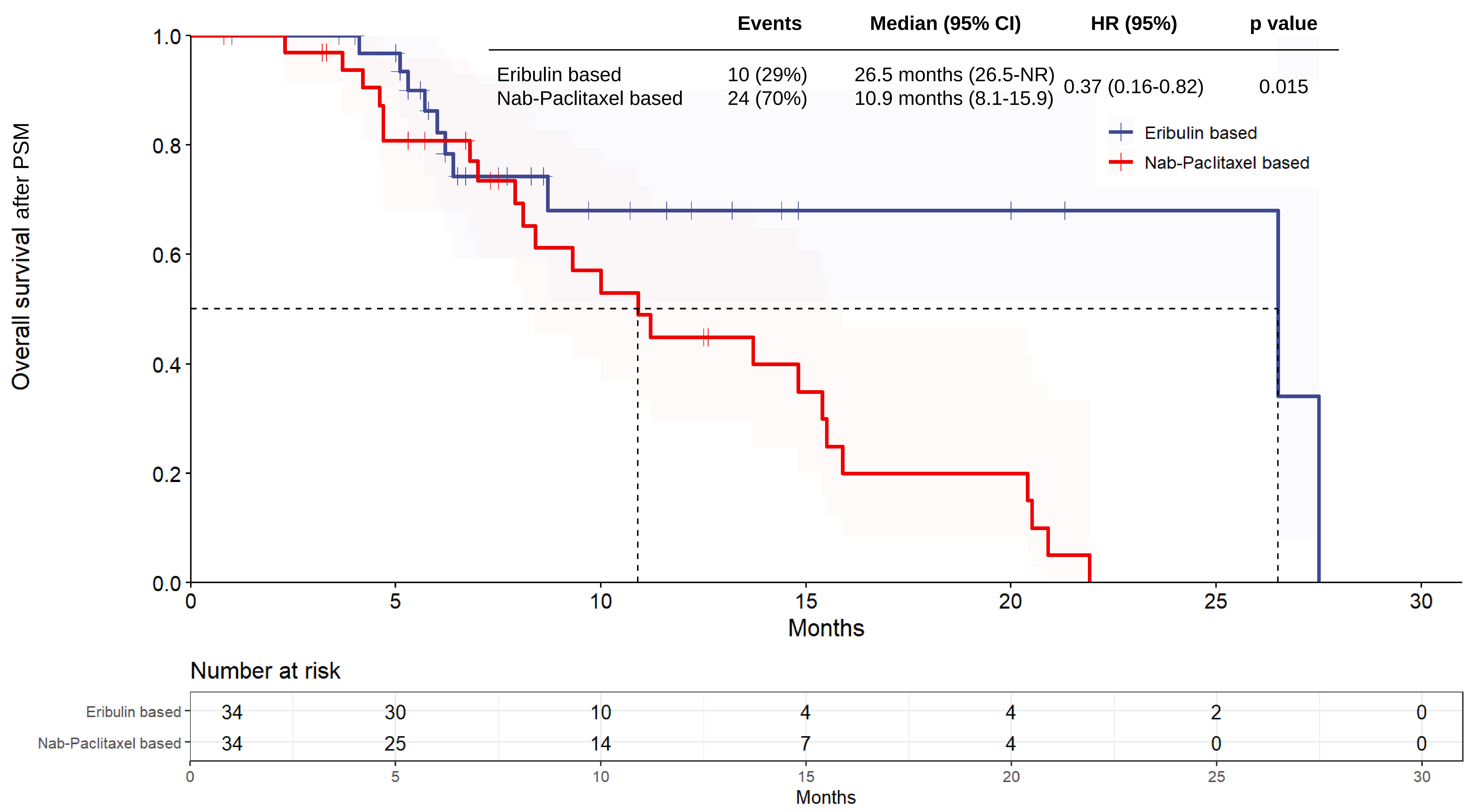
Events
Median (95% CI)
HR (95%)
p value
Eribulin based
10 (29%)
26.5 months (26.5-NR)
Nab-Paclitaxel based
24 (70%)
10.9 months (8.1-15.9)
0.37 (0.16-0.82)
0.015
Eribulin based
Nab-Paclitaxel based
Overall survival after PSM
1.0
0.8
0.6
0.4
0.2
0.0
0
5
10
15
20
25
30
Months
Number at risk
Eribulin based
34
30
10
4
4
2
0
Nab-Paclitaxel based
34
25
14
7
4
0
0
Months
