# Supplementary material for: Feasibility and tolerability of eribulin-based chemotherapy versus other chemotherapy regimens for patients with metastatic triple-negative breast cancer: a single-centre retrospective study
Source: Front Cell Dev Biol. 2024 Feb 22;12:1313610. doi: 10.3389/fcell.2024.1313610 (PMC10936577; doi:10.3389/fcell.2024.1313610)
Supplement: Supplementary file 2 [file DataSheet2.ZIP › source images/Figure 2 (EFGH). KM Analysis of PFS&OS for Eribulin and Platinum.pdf]

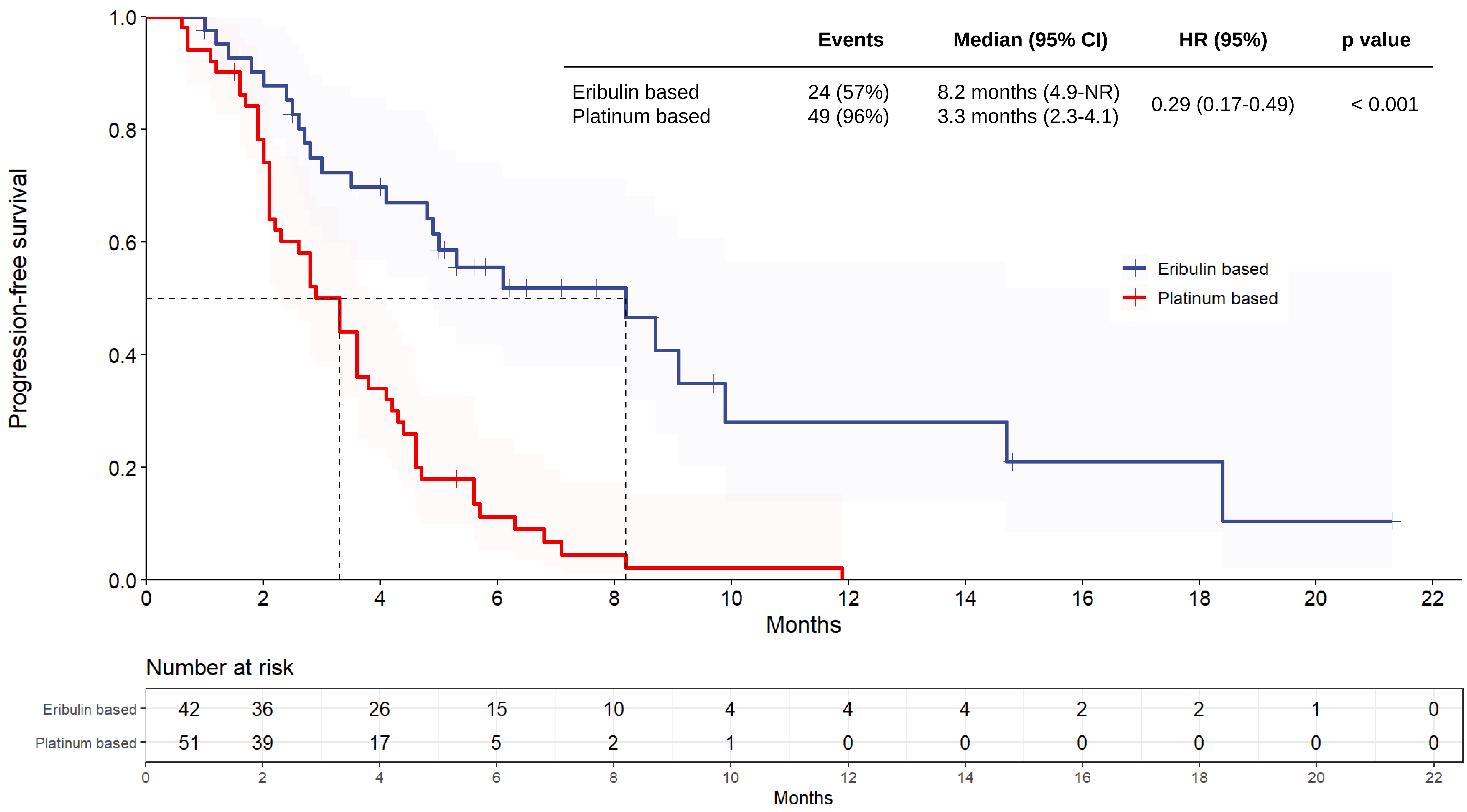
Progression-free survival
1.0
0.8
0.6
0.4
0.2
0.0
Events
Median (95% CI)
HR (95%)
p value
Eribulin based
Platinum based
24 (57%)
49 (96%)
8.2 months (4.9-NR)
3.3 months (2.3-4.1)
0.29 (0.17-0.49)
< 0.001
Eribulin based
Platinum based
0 2 4 6 8 10 12 14 16 18 20 22
Months
Number at risk
Eribulin based 42 36 26 15 10 4 4 4 2 2 1 0
Platinum based 51 39 17 5 2 1 0 0 0 0 0 0
0 2 4 6 8 10 12 14 16 18 20 22
Months

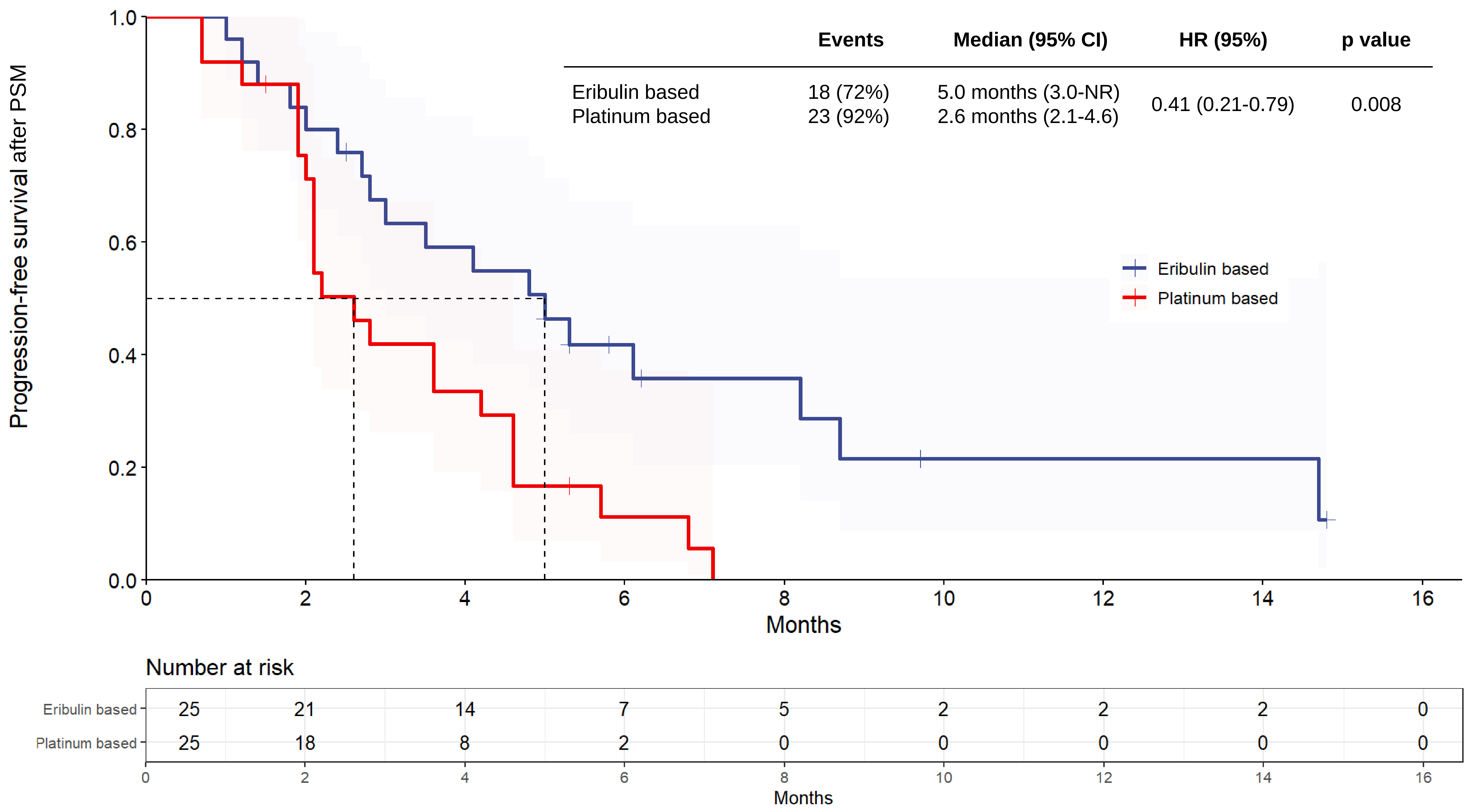

Progression-free survival after PSM
1.0
0.8
0.6
0.4
0.2
0.0
0
2
4
6
8
10
12
14
16
Months
Events
Median (95% CI)
HR (95%)
p value
Eribulin based
Platinum based
18 (72%)
23 (92%)
5.0 months (3.0-NR)
2.6 months (2.1-4.6)
0.41 (0.21-0.79)
0.008
Eribulin based
Platinum based
Number at risk
Eribulin based 25 21 14 7 5 2 2 2 0
Platinum based 25 18 8 2 0 0 0 0 0
0
2
4
6
8
10
12
14
16
Months

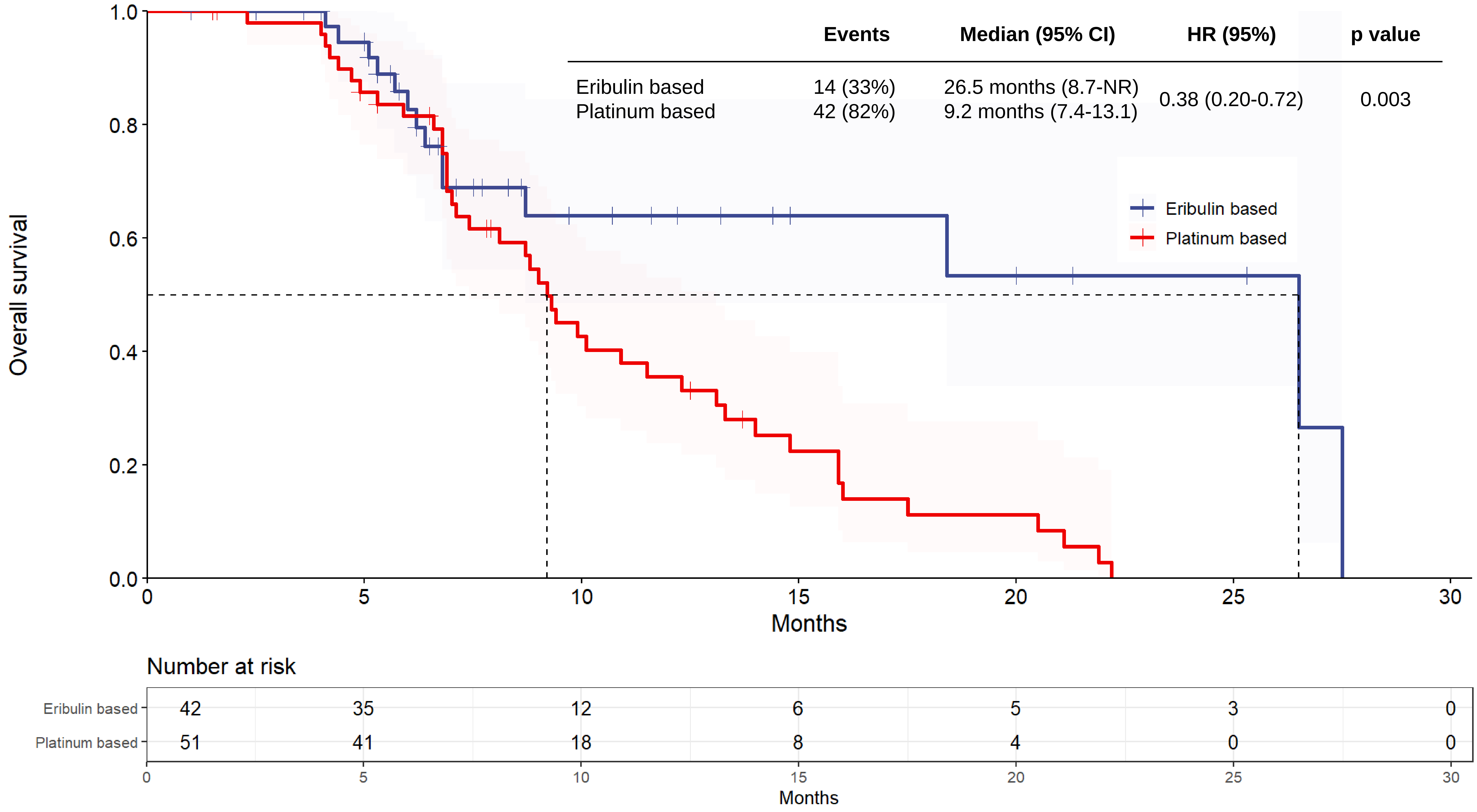
Overall survival
1.0
0.8
0.6
0.4
0.2
0.0
0
5
10
15
20
25
30
Months
Events
Median (95% CI)
HR (95%)
p value
Eribulin based
Platinum based
14 (33%)
42 (82%)
26.5 months (8.7-NR)
9.2 months (7.4-13.1)
0.38 (0.20-0.72)
0.003
Eribulin based
Platinum based
Number at risk
Eribulin based 42 35 12 6 5 3 0
Platinum based 51 41 18 8 4 0 0
Months

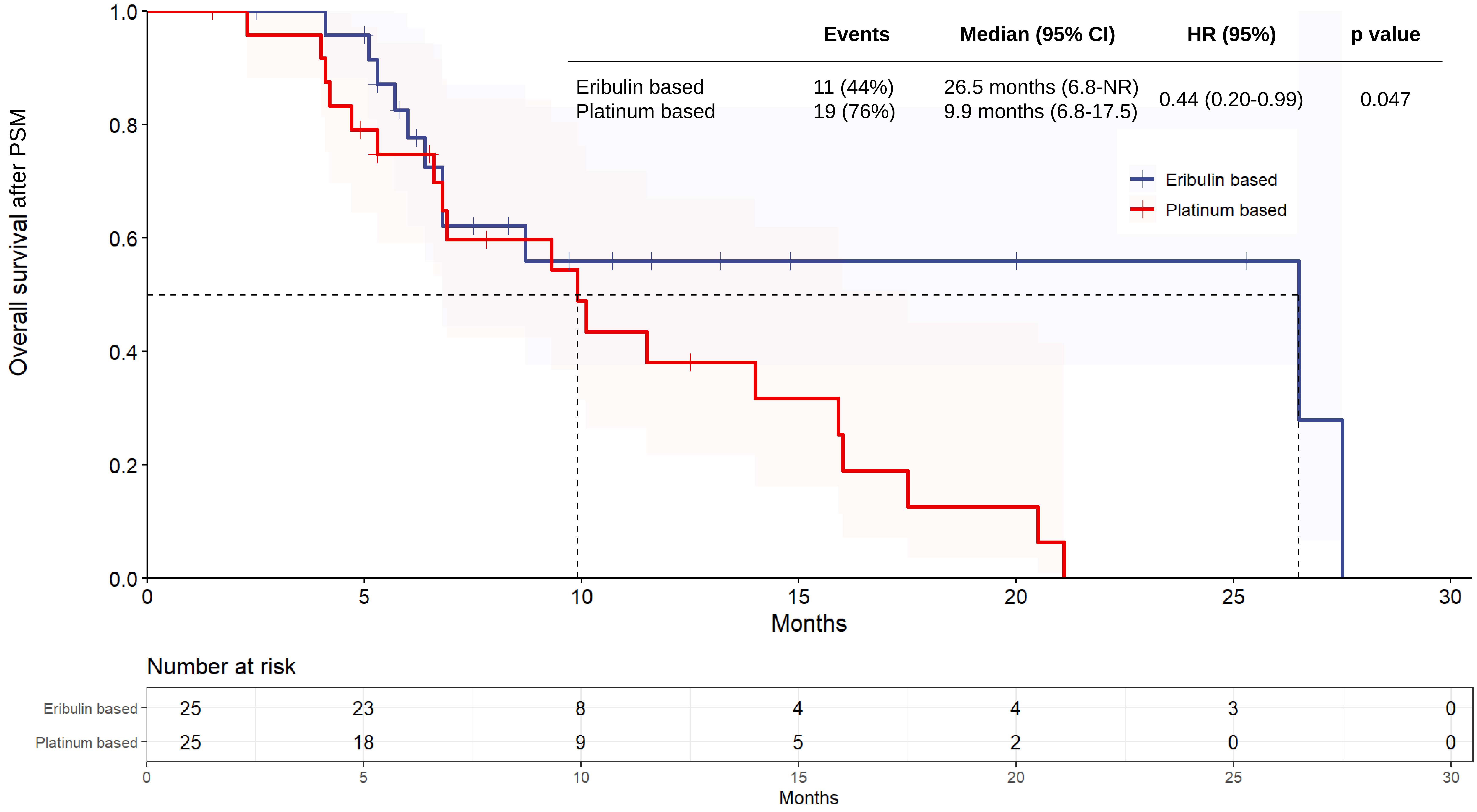

Events
Median (95% CI)
HR (95%)
p value
Eribulin based
Platinum based
11 (44%)
19 (76%)
26.5 months (6.8-NR)
9.9 months (6.8-17.5)
0.44 (0.20-0.99)
0.047
Eribulin based
Platinum based
Overall survival after PSM
1.0
0.8
0.6
0.4
0.2
0.0
0
5
10
15
20
25
30
Months
Number at risk
Eribulin based 25 23 8 4 4 3 0
Platinum based 25 18 9 5 2 0 0
Months
