# Supplementary material for: Feasibility and tolerability of eribulin-based chemotherapy versus other chemotherapy regimens for patients with metastatic triple-negative breast cancer: a single-centre retrospective study
Source: Front Cell Dev Biol. 2024 Feb 22;12:1313610. doi: 10.3389/fcell.2024.1313610 (PMC10936577; doi:10.3389/fcell.2024.1313610)
Supplement: Supplementary file 2 [file DataSheet2.ZIP › source tables/Table_15Dec2023.docx]

**Table 1.** Patient baseline demographic and clinical characteristics in the eribulin-based and NAB-paclitaxel-based groups

|  | Initial cohort | | |  | Propensity-score-matched cohort | | |
| --- | --- | --- | --- | --- | --- | --- | --- |
|  | Eribulin  Based  (n=42) | Nab-Paclitaxel  Based  (n=45) | *P* |  | Eribulin  Based  (n=34) | Nab-Paclitaxel  Based  (n=34) | *P* |
| Age, years |  |  |  |  |  |  |  |
| < 50 | 21 (50.0) | 20 (44.4) | *0.761* |  | 15 (44.1) | 14 (41.2) | *1.000* |
| ≥ 50 | 21 (50.0) | 25 (55.6) |  |  | 19 (55.9) | 20 (58.8) |  |
| ECOG PS at start |  |  |  |  |  |  |  |
| 0 | 7 (16.7) | 4 (8.9) | *0.442* |  | 4 (11.8) | 4 (11.8) | *1.000* |
| ≥ 1 | 35 (83.3) | 41 (91.1) |  |  | 30 (88.2) | 30 (88.2) |  |
| Menopausal status at diagnosis |  |  |  |  |  |  |  |
| Premenopausal | 35 (83.3) | 33 (73.3) | *0.385* |  | 27 (79.4) | 25 (73.5) | *0.775* |
| Postmenopausal | 7 (16.7) | 12 (26.7) |  |  | 7 (20.6) | 9 (26.5) |  |
| Surgery on primary tumor |  |  |  |  |  |  |  |
| Yes | 32 (76.2) | 37 (82.2) | *0.668* |  | 27 (79.4) | 27 (79.4) | *1.000* |
| No | 10 (23.8) | 8 (17.8) |  |  | 7 (20.6) | 7 (20.6) |  |
| TNBC at the initial onset |  |  |  |  |  |  |  |
| Yes | 31 (73.8) | 36 (80.0) | *0.667* |  | 26 (76.5) | 26 (76.5) | *1.000* |
| No | 11 (26.2) | 9 (20.0) |  |  | 8 (23.5) | 8 (23.5) |  |
| Ki67 ≥ 30% | 38 (90.5) | 40 (88.9) | *1.000* |  | 30 (88.2) | 29 (85.3) | *1.000* |
| Metastatic sites |  |  |  |  |  |  |  |
| Visceral | 24 (57.1) | 23 (51.1) | *0.727* |  | 16 (47.1) | 17 (50.0) | *1.000* |
| Non-visceral | 18 (42.9) | 22 (48.9) |  |  | 18 (52.9) | 17 (50.0) |  |
| Metastatic sites > 3 | 15 (35.7) | 18 (40.0) | *0.849* |  | 12 (35.3) | 11 (32.4) | *1.000* |
| Location of metastases |  |  |  |  |  |  |  |
| Brain | 1 (2.4) | 1 (2.2) | *1.000* |  | 1 (2.9) | 1 (2.9) | *1.000* |
| Bone | 16 (38.1) | 20 (44.4) | *0.702* |  | 14 (41.2) | 12 (35.3) | *0.803* |
| Liver | 9 (21.4) | 8 (17.8) | *0.874* |  | 7 (20.6) | 8 (23.5) | *1.000* |
| Lung | 19 (45.2) | 20 (44.4) | *1.000* |  | 13 (38.2) | 14 (41.2) | *1.000* |
| Lymph node | 30 (71.4) | 31 (68.9) | *0.981* |  | 25 (73.5) | 22 (64.7) | *0.600* |
| Adrenal glands | 2 (4.8) | 0 | *0.444* |  | 0 | 0 | *NA* |
| Chest wall | 7 (16.7) | 7 (15.6) | *1.000* |  | 4 (11.8) | 4 (11.8) | *1.000* |
| (Neo-) Adjuvant therapies |  |  |  |  |  |  |  |
| Paclitaxel/Docetaxel | 30 (71.4) | 29 (64.4) | *0.640* |  | 25 (73.5) | 23 (67.6) | *0.790* |
| Paclitaxel/Docetaxel and Anthracyclines | 30 (71.4) | 32 (71.1) | *1.000* |  | 9 (26.5) | 8 (23.5) | *1.000* |
| Platinum (Cis/Carbo) | 3 (7.1) | 1 (2.2) | *0.560* |  | 2 (5.9) | 1 (2.9) | *1.000* |
| Capecitabine | 8 (19.0) | 4 (8.9) | *0.288* |  | 6 (17.6) | 3 (8.8) | *0.474* |
| Treatment |  |  |  |  |  |  |  |
| Apatinib | 15 (35.7) | 13 (28.9) | *0.652* |  | 13 (38.2) | 11 (32.4) | *0.800* |
| anti-PD-1/L1 antibody | 15 (35.7) | 10 (22.2) | *0.249* |  | 13 (38.2) | 8 (23.5) | *0.294* |
| Gemcitabine | 10 (23.8) | 4 (8.9) | *0.109* |  | 7 (20.6) | 4 (11.8) | *0.510* |
| Capecitabine | 3 (7.1) | 7 (15.6) | *0.372* |  | 3 (8.8) | 3 (8.8) | *1.000* |
| Lines of therapy |  |  |  |  |  |  |  |
| 1^st^ Line | 17 (40.5) | 20 (44.4) | *0.875* |  | 13 (38.2) | 12 (35.3) | *1.000* |
| 2^nd+^ Line | 25 (59.5) | 25 (55.6) |  |  | 21 (61.8) | 22 (64.7) |  |

**Table 2**. Patient baseline demographic and clinical characteristics in eribulin-based and platinum-based groups

|  | Initial cohort | | |  | Propensity-score-matched cohort | | |
| --- | --- | --- | --- | --- | --- | --- | --- |
|  | Eribulin  Based  (n=42) | Platinum  Based  (n=51) | *P* |  | Eribulin  Based  (n=25) | Platinum  Based  (n=25) | *P* |
| Age, years |  |  |  |  |  |  |  |
| < 50 | 21 (50.0) | 26 (51.0) | *1.000* |  | 10 (40.0) | 10 (40.0) | *1.000* |
| ≥ 50 | 21 (50.0) | 25 (49.0) |  |  | 15 (60.0) | 15 (60.0) |  |
| ECOG PS at start |  |  |  |  |  |  |  |
| 0 | 7 (16.7) | 5 (9.8) | *0.502* |  | 5 (20.0) | 4 (16.0) | *1.000* |
| ≥ 1 | 35 (83.3) | 46 (90.2) |  |  | 20 (80.0) | 21 (84.0) |  |
| Menopausal status at diagnosis |  |  |  |  |  |  |  |
| Premenopausal | 35 (83.3) | 38 (74.5) | *0.437* |  | 20 (80.0) | 19 (76.0) | *1.000* |
| Postmenopausal | 7 (16.7) | 13 (25.5) |  |  | 5 (20.0) | 6 (24.0) |  |
| Surgery on primary tumor |  |  |  |  |  |  |  |
| Yes | 32 (76.2) | 36 (70.6) | *0.710* |  | 18 (72.0) | 19 (76.0) | *1.000* |
| No | 10 (23.8) | 15 (29.4) |  |  | 7 (28.0) | 6 (24.0) |  |
| TNBC at the initial onset |  |  |  |  |  |  |  |
| Yes | 31 (73.8) | 42 (82.4) | *0.457* |  | 19 (76.0) | 19 (76.0) | *1.000* |
| No | 11 (26.2) | 9 (17.6) |  |  | 6 (24.0) | 6 (24.0) |  |
| Ki67 ≥ 30% | 38 (90.5) | 44 (86.3) | *0.763* |  | 22 (88.0) | 23 (92.0) | *1.000* |
| Metastatic sites |  |  |  |  |  |  |  |
| Visceral | 24 (57.1) | 30 (58.8) | *1.000* |  | 13 (52.0) | 16 (64.0) | *0.567* |
| Non-visceral | 18 (42.9) | 21 (41.2) |  |  | 12 (48.0) | 9 (36.0) |  |
| Metastatic sites > 3 | 15 (35.7) | 26 (51.0) | *0.206* |  | 9 (36.0) | 11 (44.0) | *0.773* |
| Location of metastases |  |  |  |  |  |  |  |
| Brain | 1 (2.4) | 1 (2.0) | *1.000* |  | 0 | 0 | *1.000* |
| Bone | 16 (38.1) | 21 (41.2) | *0.929* |  | 5 (20.0) | 11 (44.0) | *0.330* |
| Liver | 9 (21.4) | 9 (17.6) | *0.845* |  | 5 (20.0) | 5 (20.0) | *1.000* |
| Lung | 19 (45.2) | 23 (45.1) | *1.000* |  | 12 (48.0) | 14 (56.0) | *0.777* |
| Lymph node | 30 (71.4) | 36 (70.6) | *1.000* |  | 17 (68.0) | 17 (68.0) | *1.000* |
| Adrenal glands | 2 (4.8) | 0 | *0.391* |  | 0 | 0 | *1.000* |
| Chest wall | 7 (16.7) | 6 (11.8) | *0.705* |  | 2 (20.0) | 6 (24.0) | *1.000* |
| (Neo-) Adjuvant therapies |  |  |  |  |  |  |  |
| Paclitaxel/Docetaxel | 30 (71.4) | 34 (66.7) | *0.788* |  | 8 (32.0) | 7 (28.0) | *1.000* |
| Paclitaxel/Docetaxel and Anthracyclines | 30 (71.4) | 40 (78.4) | *0.591* |  | 17 (68.0) | 18 (72.0) | *1.000* |
| Platinum (Cis/Carbo) | 3 (7.1) | 1 (2.0) | *0.476* |  | 2 (8.0) | 0 | *0.470* |
| Capecitabine | 8 (19.0) | 4 (7.8) | *0.196* |  | 2 (8.0) | 3 (12.0) | *1.000* |
| Treatment |  |  |  |  |  |  |  |
| Apatinib | 15 (35.7) | 0 | *<0.001* |  | 0 | 0 | *NA* |
| anti-PD-1/L1 antibody | 15 (35.7) | 1 (2.0) | *<0.001* |  | 4 (16.0) | 1 (4.0) | *0.346* |
| Gemcitabine | 10 (23.8) | 21 (41.2) | *0.122* |  | 9 (36.0) | 8 (32.0) | *1.000* |
| Capecitabine | 3 (7.1) | 1 (2.0) | *0.476* |  | 2 (8.0) | 0 | *0.470* |
| Lines of therapy |  |  |  |  |  |  |  |
| 1^st^ Line | 17 (40.5) | 19 (37.3) | *0.918* |  | 9 (36.0) | 8 (32.0) | *1.000* |
| 2^nd+^ Line | 25 (59.5) | 32 (62.7) |  |  | 16 (64.0) | 17 (68.0) |  |

**Table 3**. Patient baseline demographic and clinical characteristics in eribulin-based and other chemotherapy groups

|  | Initial cohort | | |  | Propensity-score-matched cohort | | |
| --- | --- | --- | --- | --- | --- | --- | --- |
|  | Eribulin  Based  (n=42) | Other Chemotherapy  (n=117) | *P* |  | Eribulin  Based  (n=41) | Other Chemotherapy  (n=41) | *P* |
| Age, years |  |  |  |  |  |  |  |
| < 50 | 21 (50.0) | 54 (46.2) | *0.804* |  | 21 (51.2) | 22 (53.7) | *1.000* |
| ≥ 50 | 21 (50.0) | 63 (53.8) |  |  | 20 (48.8) | 19 (46.3) |  |
| ECOG PS at start |  |  |  |  |  |  |  |
| 0 | 7 (16.7) | 9 (7.7) | *0.174* |  | 6 (14.6) | 5 (12.2) | *1.000* |
| ≥ 1 | 35 (83.3) | 108 (92.3) |  |  | 35 (85.4) | 36 (87.8) |  |
| Menopausal status at diagnosis |  |  |  |  |  |  |  |
| Premenopausal | 35 (83.3) | 85 (72.6) | *0.241* |  | 34 (82.9) | 31 (75.6) | *0.586* |
| Postmenopausal | 7 (16.7) | 32 (28.2) |  |  | 7 (17.1) | 10 (24.4) |  |
| Surgery on primary tumor |  |  |  |  |  |  |  |
| Yes | 32 (76.2) | 84 (71.8) | *0.728* |  | 31 (75.6) | 29 (70.7) | *0.803* |
| No | 10 (23.8) | 33 (28.2) |  |  | 10 (24.4) | 12 (29.3) |  |
| TNBC at the initial onset |  |  |  |  |  |  |  |
| Yes | 31 (73.8) | 89 (76.1) | *0.934* |  | 31 (75.6) | 32 (78.0) | *1.000* |
| No | 11 (26.2) | 28 (23.9) |  |  | 10 (24.4) | 9 (22.0) |  |
| Ki67 ≥ 30% | 38 (90.5) | 102 (87.2) | *0.774* |  | 37 (70.2) | 40 (97.6) | *0.356* |
| Metastatic sites |  |  |  |  |  |  |  |
| Visceral | 24 (57.1) | 62 (53.0) | *0.777* |  | 23 (56.1) | 21 (51.2) | *0.825* |
| Non-visceral | 18 (42.9) | 55 (47.0) |  |  | 18 (43.9) | 20 (48.8) |  |
| Metastatic sites > 3 | 15 (35.7) | 56 (47.9) | *0.239* |  | 14 (34.1) | 17 (41.5) | *0.649* |
| Location of metastases |  |  |  |  |  |  |  |
| Brain | 1 (2.4) | 5 (4.3) | *0.936* |  | 1 (2.4) | 1 (2.4) | *1.000* |
| Bone | 16 (38.1) | 51 (43.6) | *0.663* |  | 16 (39.0) | 17 (41.5) |  |
| Liver | 9 (21.4) | 23 (19.7) | *0.983* |  | 9 (22.0) | 7 (17.1) | *0.781* |
| Lung | 19 (45.2) | 47 (40.2) | *0.697* |  | 22 (53.7) | 23 (56.1) | *1.000* |
| Lymph node | 30 (71.4) | 83 (70.9) | *1.000* |  | 29 (70.7) | 32 (78.0) | *0.613* |
| Adrenal glands | 2 (4.8) | 2 (1.7) | *0.611* |  | 1 (2.4) | 2 (4.9) | *1.000* |
| Chest wall | 7 (16.7) | 14 (12.0) | *0.613* |  | 6 (14.6) | 6 (14.6) | *1.000* |
| (Neo-) Adjuvant therapies |  |  |  |  |  |  |  |
| Paclitaxel/Docetaxel | 30 (71.4) | 74 (63.2) | *0.443* |  | 29 (70.7) | 26 (63.4) | *0.638* |
| Paclitaxel/Docetaxel and Anthracyclines | 30 (71.4) | 83 (70.9) | *1.000* |  | 29 (70.7) | 29 (70.7) | *1.000* |
| Platinum (Cis/Carbo) | 3 (7.1) | 5 (4.3) | *0.750* |  | 3 (7.3) | 3 (7.3) | *1.000* |
| Capecitabine | 8 (19.0) | 7 (6.0) | *0.029* |  | 8 (19.5) | 3 (7.3) | *0.195* |
| Treatment |  |  |  |  |  |  |  |
| Apatinib | 15 (35.7) | 20 (17.1) | *0.023* |  | 15 (36.6) | 16 (39.0) | *1.000* |
| anti-PD-1/L1 antibody | 15 (35.7) | 12 (10.2) | *<0.001* |  | 15 (36.6) | 8 (19.5) | *0.140* |
| Gemcitabine | 10 (23.8) | 26 (22.2) | *1.000* |  | 10 (24.4) | 10 (24.4) | *1.000* |
| Capecitabine | 3 (7.1) | 24 (20.5) | *0.082* |  | 3 (7.3) | 5 (12.2) | *0.710* |
| Lines of therapy |  |  |  |  |  |  |  |
| 1^st^ Line | 17 (40.5) | 49 (41.9) | *1.000* |  | 16 (39.0) | 174 (41.5) | *1.000* |
| 2^nd+^ Line | 25 (59.5) | 68 (58.1) |  |  |  |  |  |

**Table 4. Tumor response per RECIST 1.1 (before PSM).**

|  | Eribulin  based | Nab-Paclitaxel  based | *P* |  | Platinum  based | *P* | Other  Chemotherapy | *P* |
| --- | --- | --- | --- | --- | --- | --- | --- | --- |
| **All comers, n** | 42 | 45 | *-* |  | 51 | *-* | 117 | *-* |
| ORR, n (%; 95% CI) | 21 (50.0; 34.2-65.8) | 18 (40.0; 25.7-55.7) | *0.349* |  | 14 (27.5; 15.9-41.7) | *0.026* | 37 (31.6; 23.3-40.9) | *0.034* |
| DCR, n (%; 95% CI) | 27 (64.3; 48.0-78.4) | 23 (51.1; 35.8-66.3) | *0.214* |  | 26 (51.0; 36.6-65.2) | *0.197* | 61 (52.1; 42.7-61.5) | *0.174* |
| Overall response, n (%) |  |  |  |  |  |  |  |  |
| Complete response | 0 | 0 |  |  | 0 |  | 0 |  |
| Partial response | 21 (50.0) | 18 (40.0) |  |  | 14 (27.5) |  | 37 (31.6) |  |
| Stable disease | 6 (14.3) | 5 (11.1) |  |  | 12 (23.5) |  | 24 (20.5) |  |
| Progressive disease | 15 (35.7) | 20 (44.4) |  |  | 24 (47.1) |  | 52 (44.4) |  |
| Not evaluable | 0 | 2 (4.4) |  |  | 1 (2.0) |  | 4 (3.4) |  |
| **1^st^ line, n** | 17 | 20 | *-* |  | 19 | *-* | 49 | *-* |
| ORR, n (%; 95% CI) | 11 (64.7; 38.3-85.8) | 9 (45.0; 23.1-68.5) | *0.456* |  | 6 (31.6; 12.6-56.6) | *0.262* | 19 (38.8; 25.2-53.8) | *0.064* |
| DCR, n (%; 95% CI) | 13 (76.5; 50.1-93.2) | 12 (60.0; 36.1-80.9) | *-* |  | 11 (57.9; 33.5-79.7) | *-* | 30 (61.2; 46.2-74.8) | *-* |
| Overall response, n (%) |  |  |  |  |  |  |  |  |
| Complete response | 0 | 0 |  |  | 0 |  | 0 |  |
| Partial response | 11 (64.7) | 9 (45.0) |  |  | 6 (31.6) |  | 19 (38.8) |  |
| Stable disease | 2 (11.8) | 3 (15.0) |  |  | 5 (26.3) |  | 11 (22.4) |  |
| Progressive disease | 4 (23.5) | 6 (30.0) |  |  | 7 (36.8) |  | 15 (30.6) |  |
| Not evaluable | 0 | 2 (10.0) |  |  | 1 (5.3) |  | 4 (8.2) |  |
| **2^nd+^ line, n** | 25 | 25 | *-* |  | 32 | *-* | 68 | *-* |
| ORR, n (%; 95% CI) | 10 (40.0; 21.1-61.3) | 9 (36.0; 18.0-57.5) | *0.771* |  | 8 (25.0; 11.5-43.4) | *0.227* | 18 (26.4; 16.5-38.6) | *0.207* |
| DCR, n (%; 95% CI) | 14 (56.0; 34.9-75.6) | 11 (44.0; 24.4-65.1) | *-* |  | 15 (46.9; 29.1-65.3) | *-* | 31 (45.6; 33.5-58.1) | *-* |
| Overall response, n (%) |  |  |  |  |  |  |  |  |
| Complete response | 0 | 0 |  |  | 0 |  | 0 |  |
| Partial response | 10 (40.0) | 9 (36.0) |  |  | 8 (25.0) |  | 18 (26.4) |  |
| Stable disease | 4 (16.0) | 2 (8.0) |  |  | 7 (21.9) |  | 13 (19.1) |  |
| Progressive disease | 11 (44.0) | 14 (56.0) |  |  | 17 (53.1) |  | 37 (54.4) |  |
| Not evaluable | 0 | 0 |  |  | 0 |  | 0 |  |

**Table 5. Tumor response per RECIST 1.1 (after PSM).**

|  | Eribulin  based | Nab-Paclitaxel based | *P* |  | Eribulin  based | Platinum  based | *P* | Eribulin  based | Other Chemotherapy | *P* |
| --- | --- | --- | --- | --- | --- | --- | --- | --- | --- | --- |
| **All comers, n** | 34 | 34 |  |  | 25 | 25 | *-* | 41 | 41 | *-* |
| ORR, n (%; 95% CI) | 17 (50.0; 32.4-67.6)) | 14 (41.2; 24.6-59.3) | *0.626* |  | 9 (36.0; 18.0-57.5) | 5 (20.0; 6.8-40.7) | *0.208* | 20 (48.8; 32.9-64.9) | 15 (36.6; 22.1-53.1) | *0.264* |
| DCR, n (%; 95% CI) | 23 (67.6; 49.5-82.6) | 19 (55.9; 37.9-72.8) | *0.454* |  | 12 (48.0; 27.8-68.7) | 12 (48.0; 27.8-68.7) | *1.000* | 26 (63.4; 46.9-77.9) | 23 (56.1; 39.7-71.5) | *0.499* |
| Overall response, n (%) |  |  |  |  |  |  |  |  |  |  |
| Complete response | 0 | 0 |  |  | 0 | 0 |  | 0 | 0 |  |
| Partial response | 17 (50.0) | 14 (41.2) |  |  | 9 (36.0) | 5 (20.0) |  | 20 (48.8) | 15 (36.6) |  |
| Stable disease | 6 (17.6) | 5 (14.7) |  |  | 3 (12.0) | 7 (28.0) |  | 6 (14.6) | 8 (19.5) |  |
| Progressive disease | 11 (32.4) | 14 (41.2) |  |  | 13 (52.0) | 13 (52.0) |  | 15 (36.6) | 16 (39.0) |  |
| Not evaluable | 0 | 1 (2.9) |  |  | 0 | 0 |  | 0 | 2 (49) |  |
| **1^st^ line, n** | 13 | 12 | *-* |  | 9 | 8 | *-* | 16 | 17 | *-* |
| ORR, n (%; 95% CI) | 9 (69.2; 38.6-90.9) | 5 (41.7; 15.2-72.3) | *0.333* |  | 4 (44.4; 13.7-78.8) | 2 (25.0; 3.2-65.1) | *0.439* | 10 (62.5; 35.4-84.8) | 8 (47.1; 23.0-72.2) | *0.544* |
| DCR, n (%; 95% CI) | 11 (84.6; 54.6-98.1) | 8 (66.7; 34.9-90.1) | *-* |  | 6 (66.7; 30.0-92.5) | 5 (62.5; 24.5-91.5) | *-* | 12 (75.0; 47.6-92.7) | 11 (64.7; 38.3-85.8) | *-* |
| Overall response, n (%) |  |  |  |  |  |  |  |  |  |  |
| Complete response | 0 | 0 |  |  | 0 | 0 |  | 0 | 0 |  |
| Partial response | 9 (69.2) | 5 (41.7) |  |  | 4 (44.4) | 2 (25.0) |  | 10 (62.5) | 8 (47.1) |  |
| Stable disease | 2 (15.4) | 3 (25.0) |  |  | 2 (22.2) | 3 (37.5) |  | 2 (12.5) | 3 (17.6) |  |
| Progressive disease | 2 (15.4) | 3 (25.0) |  |  | 3 (33.3) | 3 (37.5) |  | 4 (25.0) | 4 (23.5) |  |
| Not evaluable | 0 | 1 (8.3) |  |  | 0 | 0 |  | 0 | 2 (11.8) |  |
| **2^nd+^ line, n** | 21 | 22 | *-* |  | 16 | 17 | *-* | 25 | 24 | *-* |
| ORR, n (%; 95% CI) | 8 (38.1; 18.1-61.6) | 9 (40.9; 20.7-63.6) | *0.850* |  | 5 (31.3; 11.0-58.7) | 3 (17.6; 3.8-43.4) | *0.482* | 10 (40.0; 21.1-61.3) | 7 (29.2; 12.6-51.1) | *0.426* |
| DCR, n (%; 95% CI) | 12 (57.1; 34.0-78.2) | 11 (50.0; 28.2-71.8) | *-* |  | 6 (37.5; 15.2-64.6) | 7 (41.2; 18.4-67.1) | *-* | 14 (56.0; 34.9-75.6) | 12 (50.0; 29.1-70.9) | *-* |
| Overall response, n (%) |  |  |  |  |  |  |  |  |  |  |
| Complete response | 0 | 0 |  |  | 0 | 0 |  | 0 | 0 |  |
| Partial response | 8 (38.1) | 9 (40.9) |  |  | 5 (31.3) | 3 (17.7) |  | 10 (40.0) | 7 (29.2) |  |
| Stable disease | 4 (19.0) | 2 (9.1) |  |  | 1 (6.2) | 4 (23.5) |  | 4 (16.0) | 5 (20.8) |  |
| Progressive disease | 9 (42.9) | 11 (50.0) |  |  | 10 (62.5) | 10 (58.8) |  | 11 (44.0) | 12 (50.0) |  |
| Not evaluable | 0 | 0 |  |  | 0 | 0 |  | 0 | 0 |  |
