# Supplementary material for: Feasibility and tolerability of eribulin-based chemotherapy versus other chemotherapy regimens for patients with metastatic triple-negative breast cancer: a single-centre retrospective study
Source: Front Cell Dev Biol. 2024 Feb 22;12:1313610. doi: 10.3389/fcell.2024.1313610 (PMC10936577; doi:10.3389/fcell.2024.1313610)
Supplement: Supplementary file 2 [file DataSheet2.ZIP › source tables/Table_15Dec2023.pdf]

Table 1. Patient baseline demographic and clinical characteristics in the eribulin-based and NAB-paclitaxel-based groups

	Initial cohort			Propensity-score-matched cohort		
	Eribulin Based (n=42)	Nab-Paclitaxel Based (n=45)	*P*	Eribulin Based (n=34)	Nab-Paclitaxel Based (n=34)	*P*
Age, years						
< 50	21 (50.0)	20 (44.4)	*0.761*	15 (44.1)	14 (41.2)	*1.000*
≥ 50	21 (50.0)	25 (55.6)		19 (55.9)	20 (58.8)	
ECOG PS at start						
0	7 (16.7)	4 (8.9)	*0.442*	4 (11.8)	4 (11.8)	*1.000*
≥ 1	35 (83.3)	41 (91.1)		30 (88.2)	30 (88.2)	
Menopausal status at diagnosis						
Premenopausal	35 (83.3)	33 (73.3)	*0.385*	27 (79.4)	25 (73.5)	*0.775*
Postmenopausal	7 (16.7)	12 (26.7)		7 (20.6)	9 (26.5)	
Surgery on primary tumor						
Yes	32 (76.2)	37 (82.2)	*0.668*	27 (79.4)	27 (79.4)	*1.000*
No	10 (23.8)	8 (17.8)		7 (20.6)	7 (20.6)	
TNBC at the initial onset						
Yes	31 (73.8)	36 (80.0)	*0.667*	26 (76.5)	26 (76.5)	*1.000*
No	11 (26.2)	9 (20.0)		8 (23.5)	8 (23.5)	
Ki67 ≥ 30%	38 (90.5)	40 (88.9)	*1.000*	30 (88.2)	29 (85.3)	*1.000*
Metastatic sites						
Visceral	24 (57.1)	23 (51.1)	*0.727*	16 (47.1)	17 (50.0)	*1.000*
Non-visceral	18 (42.9)	22 (48.9)		18 (52.9)	17 (50.0)	
Metastatic sites > 3	15 (35.7)	18 (40.0)	*0.849*	12 (35.3)	11 (32.4)	*1.000*
Location of metastases						
Brain	1 (2.4)	1 (2.2)	*1.000*	1 (2.9)	1 (2.9)	*1.000*
Bone	16 (38.1)	20 (44.4)	*0.702*	14 (41.2)	12 (35.3)	*0.803*
Liver	9 (21.4)	8 (17.8)	*0.874*	7 (20.6)	8 (23.5)	*1.000*
Lung	19 (45.2)	20 (44.4)	*1.000*	13 (38.2)	14 (41.2)	*1.000*
Lymph node	30 (71.4)	31 (68.9)	*0.981*	25 (73.5)	22 (64.7)	*0.600*
Adrenal glands	2 (4.8)	0	*0.444*	0	0	*NA*
Chest wall	7 (16.7)	7 (15.6)	*1.000*	4 (11.8)	4 (11.8)	*1.000*
(Neo-) Adjuvant therapies						
Paclitaxel/Docetaxel	30 (71.4)	29 (64.4)	*0.640*	25 (73.5)	23 (67.6)	*0.790*
Paclitaxel/Docetaxel and Anthracyclines	30 (71.4)	32 (71.1)	*1.000*	9 (26.5)	8 (23.5)	*1.000*
Platinum (Cis/Carbo)	3 (7.1)	1 (2.2)	*0.560*	2 (5.9)	1 (2.9)	*1.000*
Capecitabine	8 (19.0)	4 (8.9)	*0.288*	6 (17.6)	3 (8.8)	*0.474*
Treatment						
Apatinib	15 (35.7)	13 (28.9)	*0.652*	13 (38.2)	11 (32.4)	*0.800*
anti-PD-1/L1 antibody	15 (35.7)	10 (22.2)	*0.249*	13 (38.2)	8 (23.5)	*0.294*
Gemcitabine	10 (23.8)	4 (8.9)	*0.109*	7 (20.6)	4 (11.8)	*0.510*
Capecitabine	3 (7.1)	7 (15.6)	*0.372*	3 (8.8)	3 (8.8)	*1.000*
Lines of therapy						
1st Line	17 (40.5)	20 (44.4)	*0.875*	13 (38.2)	12 (35.3)	*1.000*
2^{nd+} Line	25 (59.5)	25 (55.6)		21 (61.8)	22 (64.7)	

Table 2. Patient baseline demographic and clinical characteristics in eribulin-based and platinum-based groups

	Initial cohort			Propensity-score-matched cohort		
	Eribulin Based (n=42)	Platinum Based (n=51)	*P*	Eribulin Based (n=25)	Platinum Based (n=25)	*P*
Age, years						
< 50	21 (50.0)	26 (51.0)	*1.000*	10 (40.0)	10 (40.0)	*1.000*
≥ 50	21 (50.0)	25 (49.0)		15 (60.0)	15 (60.0)	
ECOG PS at start						
0	7 (16.7)	5 (9.8)	*0.502*	5 (20.0)	4 (16.0)	*1.000*
≥ 1	35 (83.3)	46 (90.2)		20 (80.0)	21 (84.0)	
Menopausal status at diagnosis						
Premenopausal	35 (83.3)	38 (74.5)	*0.437*	20 (80.0)	19 (76.0)	*1.000*
Postmenopausal	7 (16.7)	13 (25.5)		5 (20.0)	6 (24.0)	
Surgery on primary tumor						
Yes	32 (76.2)	36 (70.6)	*0.710*	18 (72.0)	19 (76.0)	*1.000*
No	10 (23.8)	15 (29.4)		7 (28.0)	6 (24.0)	
TNBC at the initial onset						
Yes	31 (73.8)	42 (82.4)	*0.457*	19 (76.0)	19 (76.0)	*1.000*
No	11 (26.2)	9 (17.6)		6 (24.0)	6 (24.0)	
Ki67 ≥ 30%	38 (90.5)	44 (86.3)	*0.763*	22 (88.0)	23 (92.0)	*1.000*
Metastatic sites						
Visceral	24 (57.1)	30 (58.8)	*1.000*	13 (52.0)	16 (64.0)	*0.567*
Non-visceral	18 (42.9)	21 (41.2)		12 (48.0)	9 (36.0)	
Metastatic sites > 3	15 (35.7)	26 (51.0)	*0.206*	9 (36.0)	11 (44.0)	*0.773*
Location of metastases						
Brain	1 (2.4)	1 (2.0)	*1.000*	0	0	*1.000*
Bone	16 (38.1)	21 (41.2)	*0.929*	5 (20.0)	11 (44.0)	*0.330*
Liver	9 (21.4)	9 (17.6)	*0.845*	5 (20.0)	5 (20.0)	*1.000*
Lung	19 (45.2)	23 (45.1)	*1.000*	12 (48.0)	14 (56.0)	*0.777*
Lymph node	30 (71.4)	36 (70.6)	*1.000*	17 (68.0)	17 (68.0)	*1.000*
Adrenal glands	2 (4.8)	0	*0.391*	0	0	*1.000*
Chest wall	7 (16.7)	6 (11.8)	*0.705*	2 (20.0)	6 (24.0)	*1.000*
(Neo-) Adjuvant therapies						
Paclitaxel/Docetaxel	30 (71.4)	34 (66.7)	*0.788*	8 (32.0)	7 (28.0)	*1.000*
Paclitaxel/Docetaxel and Anthracyclines	30 (71.4)	40 (78.4)	*0.591*	17 (68.0)	18 (72.0)	*1.000*
Platinum (Cis/Carbo)	3 (7.1)	1 (2.0)	*0.476*	2 (8.0)	0	*0.470*
Capecitabine	8 (19.0)	4 (7.8)	*0.196*	2 (8.0)	3 (12.0)	*1.000*
Treatment						
Apatinib	15 (35.7)	0	*<0.001*	0	0	*NA*
anti-PD-1/L1 antibody	15 (35.7)	1 (2.0)	*<0.001*	4 (16.0)	1 (4.0)	*0.346*
Gemcitabine	10 (23.8)	21 (41.2)	*0.122*	9 (36.0)	8 (32.0)	*1.000*
Capecitabine	3 (7.1)	1 (2.0)	*0.476*	2 (8.0)	0	*0.470*
Lines of therapy						
1st Line	17 (40.5)	19 (37.3)	*0.918*	9 (36.0)	8 (32.0)	*1.000*
2^{nd+} Line	25 (59.5)	32 (62.7)		16 (64.0)	17 (68.0)	

Table 3. Patient baseline demographic and clinical characteristics in eribulin-based and other chemotherapy groups

	Initial cohort			Propensity-score-matched cohort		
	Eribulin Based (n=42)	Other Chemotherapy (n=117)	*P*	Eribulin Based (n=41)	Other Chemotherapy (n=41)	*P*
Age, years						
< 50	21 (50.0)	54 (46.2)	*0.804*	21 (51.2)	22 (53.7)	*1.000*
≥ 50	21 (50.0)	63 (53.8)		20 (48.8)	19 (46.3)	
ECOG PS at start						
0	7 (16.7)	9 (7.7)	*0.174*	6 (14.6)	5 (12.2)	*1.000*
≥ 1	35 (83.3)	108 (92.3)		35 (85.4)	36 (87.8)	
Menopausal status at diagnosis						
Premenopausal	35 (83.3)	85 (72.6)	*0.241*	34 (82.9)	31 (75.6)	*0.586*
Postmenopausal	7 (16.7)	32 (28.2)		7 (17.1)	10 (24.4)	
Surgery on primary tumor						
Yes	32 (76.2)	84 (71.8)	*0.728*	31 (75.6)	29 (70.7)	*0.803*
No	10 (23.8)	33 (28.2)		10 (24.4)	12 (29.3)	
TNBC at the initial onset						
Yes	31 (73.8)	89 (76.1)	*0.934*	31 (75.6)	32 (78.0)	*1.000*
No	11 (26.2)	28 (23.9)		10 (24.4)	9 (22.0)	
Ki67 ≥ 30%	38 (90.5)	102 (87.2)	*0.774*	37 (70.2)	40 (97.6)	*0.356*
Metastatic sites						
Visceral	24 (57.1)	62 (53.0)	*0.777*	23 (56.1)	21 (51.2)	*0.825*
Non-visceral	18 (42.9)	55 (47.0)		18 (43.9)	20 (48.8)	
Metastatic sites > 3	15 (35.7)	56 (47.9)	*0.239*	14 (34.1)	17 (41.5)	*0.649*
Location of metastases						
Brain	1 (2.4)	5 (4.3)	*0.936*	1 (2.4)	1 (2.4)	*1.000*
Bone	16 (38.1)	51 (43.6)	*0.663*	16 (39.0)	17 (41.5)	
Liver	9 (21.4)	23 (19.7)	*0.983*	9 (22.0)	7 (17.1)	*0.781*
Lung	19 (45.2)	47 (40.2)	*0.697*	22 (53.7)	23 (56.1)	*1.000*
Lymph node	30 (71.4)	83 (70.9)	*1.000*	29 (70.7)	32 (78.0)	*0.613*
Adrenal glands	2 (4.8)	2 (1.7)	*0.611*	1 (2.4)	2 (4.9)	*1.000*
Chest wall	7 (16.7)	14 (12.0)	*0.613*	6 (14.6)	6 (14.6)	*1.000*
(Neo-) Adjuvant therapies						
Paclitaxel/Docetaxel	30 (71.4)	74 (63.2)	*0.443*	29 (70.7)	26 (63.4)	*0.638*
Paclitaxel/Docetaxel and Anthracyclines	30 (71.4)	83 (70.9)	*1.000*	29 (70.7)	29 (70.7)	*1.000*
Platinum (Cis/Carbo)	3 (7.1)	5 (4.3)	*0.750*	3 (7.3)	3 (7.3)	*1.000*
Capecitabine	8 (19.0)	7 (6.0)	*0.029*	8 (19.5)	3 (7.3)	*0.195*
Treatment						
Apatinib	15 (35.7)	20 (17.1)	*0.023*	15 (36.6)	16 (39.0)	*1.000*
anti-PD-1/L1 antibody	15 (35.7)	12 (10.2)	*<0.001*	15 (36.6)	8 (19.5)	*0.140*
Gemcitabine	10 (23.8)	26 (22.2)	*1.000*	10 (24.4)	10 (24.4)	*1.000*
Capecitabine	3 (7.1)	24 (20.5)	*0.082*	3 (7.3)	5 (12.2)	*0.710*
Lines of therapy						
1st Line	17 (40.5)	49 (41.9)	*1.000*	16 (39.0)	174 (41.5)	*1.000*
2^{nd+} Line	25 (59.5)	68 (58.1)				

Table 4. Tumor response per RECIST 1.1 (before PSM).

	Eribulin based	Nab-Paclitaxel based	*P*	Platinum based	*P*	Other Chemotherapy	*P*
**All comers, n**	42	45	-	51	-	117	-
ORR, n (%; 95% CI)	21 (50.0; 34.2-65.8)	18 (40.0; 25.7-55.7)	*0.349*	14 (27.5; 15.9-41.7)	*0.026*	37 (31.6; 23.3-40.9)	*0.034*
DCR, n (%; 95% CI)	27 (64.3; 48.0-78.4)	23 (51.1; 35.8-66.3)	*0.214*	26 (51.0; 36.6-65.2)	*0.197*	61 (52.1; 42.7-61.5)	*0.174*
Overall response, n (%)							
Complete response	0	0		0		0	
Partial response	21 (50.0)	18 (40.0)		14 (27.5)		37 (31.6)	
Stable disease	6 (14.3)	5 (11.1)		12 (23.5)		24 (20.5)	
Progressive disease	15 (35.7)	20 (44.4)		24 (47.1)		52 (44.4)	
Not evaluable	0	2 (4.4)		1 (2.0)		4 (3.4)	
**1st line, n**	17	20	-	19	-	49	-
ORR, n (%; 95% CI)	11 (64.7; 38.3-85.8)	9 (45.0; 23.1-68.5)	*0.456*	6 (31.6; 12.6-56.6)	*0.262*	19 (38.8; 25.2-53.8)	*0.064*
DCR, n (%; 95% CI)	13 (76.5; 50.1-93.2)	12 (60.0; 36.1-80.9)	-	11 (57.9; 33.5-79.7)	-	30 (61.2; 46.2-74.8)	-
Overall response, n (%)							
Complete response	0	0		0		0	
Partial response	11 (64.7)	9 (45.0)		6 (31.6)		19 (38.8)	
Stable disease	2 (11.8)	3 (15.0)		5 (26.3)		11 (22.4)	
Progressive disease	4 (23.5)	6 (30.0)		7 (36.8)		15 (30.6)	
Not evaluable	0	2 (10.0)		1 (5.3)		4 (8.2)	
**2nd+ line, n**	25	25	-	32	-	68	-
ORR, n (%; 95% CI)	10 (40.0; 21.1-61.3)	9 (36.0; 18.0-57.5)	*0.771*	8 (25.0; 11.5-43.4)	*0.227*	18 (26.4; 16.5-38.6)	*0.207*
DCR, n (%; 95% CI)	14 (56.0; 34.9-75.6)	11 (44.0; 24.4-65.1)	-	15 (46.9; 29.1-65.3)	-	31 (45.6; 33.5-58.1)	-
Overall response, n (%)							
Complete response	0	0		0		0	
Partial response	10 (40.0)	9 (36.0)		8 (25.0)		18 (26.4)	
Stable disease	4 (16.0)	2 (8.0)		7 (21.9)		13 (19.1)	
Progressive disease	11 (44.0)	14 (56.0)		17 (53.1)		37 (54.4)	
Not evaluable	0	0		0		0	

Table 5. Tumor response per RECIST 1.1 (after PSM).

	Eribulin based	Nab-Paclitaxel based	*P*	Eribulin based	Platinum based	*P*	Eribulin based	Other Chemotherapy	*P*
**All comers, n**	34	34		25	25	-	41	41	-
ORR, n (%; 95% CI)	17 (50.0; 32.4-67.6))	14 (41.2; 24.6-59.3)	*0.626*	9 (36.0; 18.0-57.5)	5 (20.0; 6.8-40.7)	*0.208*	20 (48.8; 32.9-64.9)	15 (36.6; 22.1-53.1)	*0.264*
DCR, n (%; 95% CI)	23 (67.6; 49.5-82.6)	19 (55.9; 37.9-72.8)	*0.454*	12 (48.0; 27.8-68.7)	12 (48.0; 27.8-68.7)	*1.000*	26 (63.4; 46.9-77.9)	23 (56.1; 39.7-71.5)	*0.499*
Overall response, n (%)									
Complete response	0	0		0	0		0	0	
Partial response	17 (50.0)	14 (41.2)		9 (36.0)	5 (20.0)		20 (48.8)	15 (36.6)	
Stable disease	6 (17.6)	5 (14.7)		3 (12.0)	7 (28.0)		6 (14.6)	8 (19.5)	
Progressive disease	11 (32.4)	14 (41.2)		13 (52.0)	13 (52.0)		15 (36.6)	16 (39.0)	
Not evaluable	0	1 (2.9)		0	0		0	2 (49)	
**1st line, n**	13	12	-	9	8	-	16	17	-
ORR, n (%; 95% CI)	9 (69.2; 38.6-90.9)	5 (41.7; 15.2-72.3)	*0.333*	4 (44.4; 13.7-78.8)	2 (25.0; 3.2-65.1)	*0.439*	10 (62.5; 35.4-84.8)	8 (47.1; 23.0-72.2)	*0.544*
DCR, n (%; 95% CI)	11 (84.6; 54.6-98.1)	8 (66.7; 34.9-90.1)	-	6 (66.7; 30.0-92.5)	5 (62.5; 24.5-91.5)	-	12 (75.0; 47.6-92.7)	11 (64.7; 38.3-85.8)	-
Overall response, n (%)									
Complete response	0	0		0	0		0	0	
Partial response	9 (69.2)	5 (41.7)		4 (44.4)	2 (25.0)		10 (62.5)	8 (47.1)	
Stable disease	2 (15.4)	3 (25.0)		2 (22.2)	3 (37.5)		2 (12.5)	3 (17.6)	
Progressive disease	2 (15.4)	3 (25.0)		3 (33.3)	3 (37.5)		4 (25.0)	4 (23.5)	
Not evaluable	0	1 (8.3)		0	0		0	2 (11.8)	
**2nd+ line, n**	21	22	-	16	17	-	25	24	-
ORR, n (%; 95% CI)	8 (38.1; 18.1-61.6)	9 (40.9; 20.7-63.6)	*0.850*	5 (31.3; 11.0-58.7)	3 (17.6; 3.8-43.4)	*0.482*	10 (40.0; 21.1-61.3)	7 (29.2; 12.6-51.1)	*0.426*
DCR, n (%; 95% CI)	12 (57.1; 34.0-78.2)	11 (50.0; 28.2-71.8)	-	6 (37.5; 15.2-64.6)	7 (41.2; 18.4-67.1)	-	14 (56.0; 34.9-75.6)	12 (50.0; 29.1-70.9)	-
Overall response, n (%)									
Complete response	0	0		0	0		0	0	
Partial response	8 (38.1)	9 (40.9)		5 (31.3)	3 (17.7)		10 (40.0)	7 (29.2)	
Stable disease	4 (19.0)	2 (9.1)		1 (6.2)	4 (23.5)		4 (16.0)	5 (20.8)	
Progressive disease	9 (42.9)	11 (50.0)		10 (62.5)	10 (58.8)		11 (44.0)	12 (50.0)	
Not evaluable	0	0		0	0		0	0	
