# Supplementary figures and images for: Feasibility and tolerability of eribulin-based chemotherapy versus other chemotherapy regimens for patients with metastatic triple-negative breast cancer: a single-centre retrospective study
Source: Front Cell Dev Biol. 2024 Feb 22;12:1313610. doi: 10.3389/fcell.2024.1313610 (PMC10936577; doi:10.3389/fcell.2024.1313610)

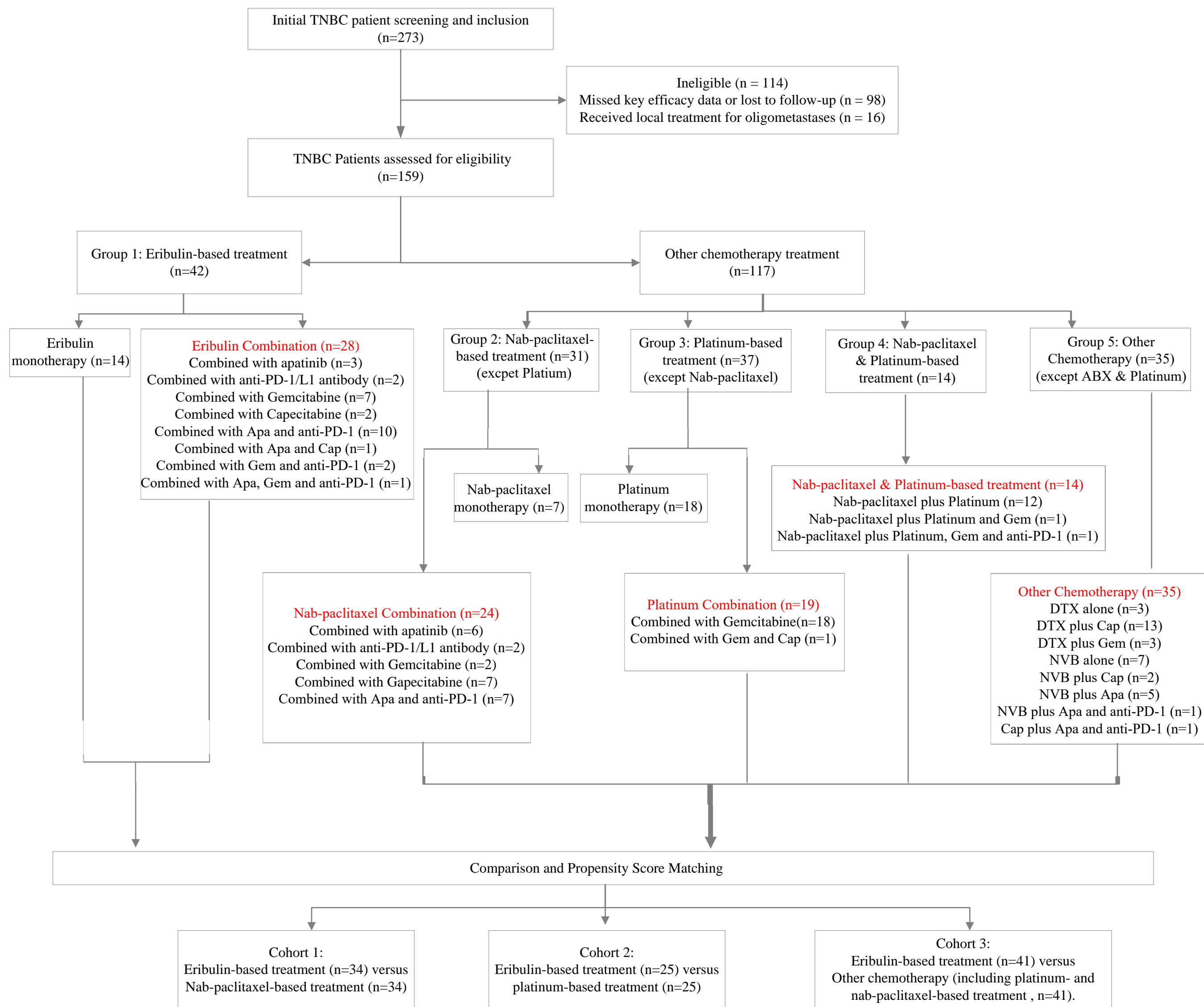

Supplement: Supplementary file 2 [file DataSheet2.ZIP › source images/Figure 1. Patient flow chart.pdf]

Overall survival

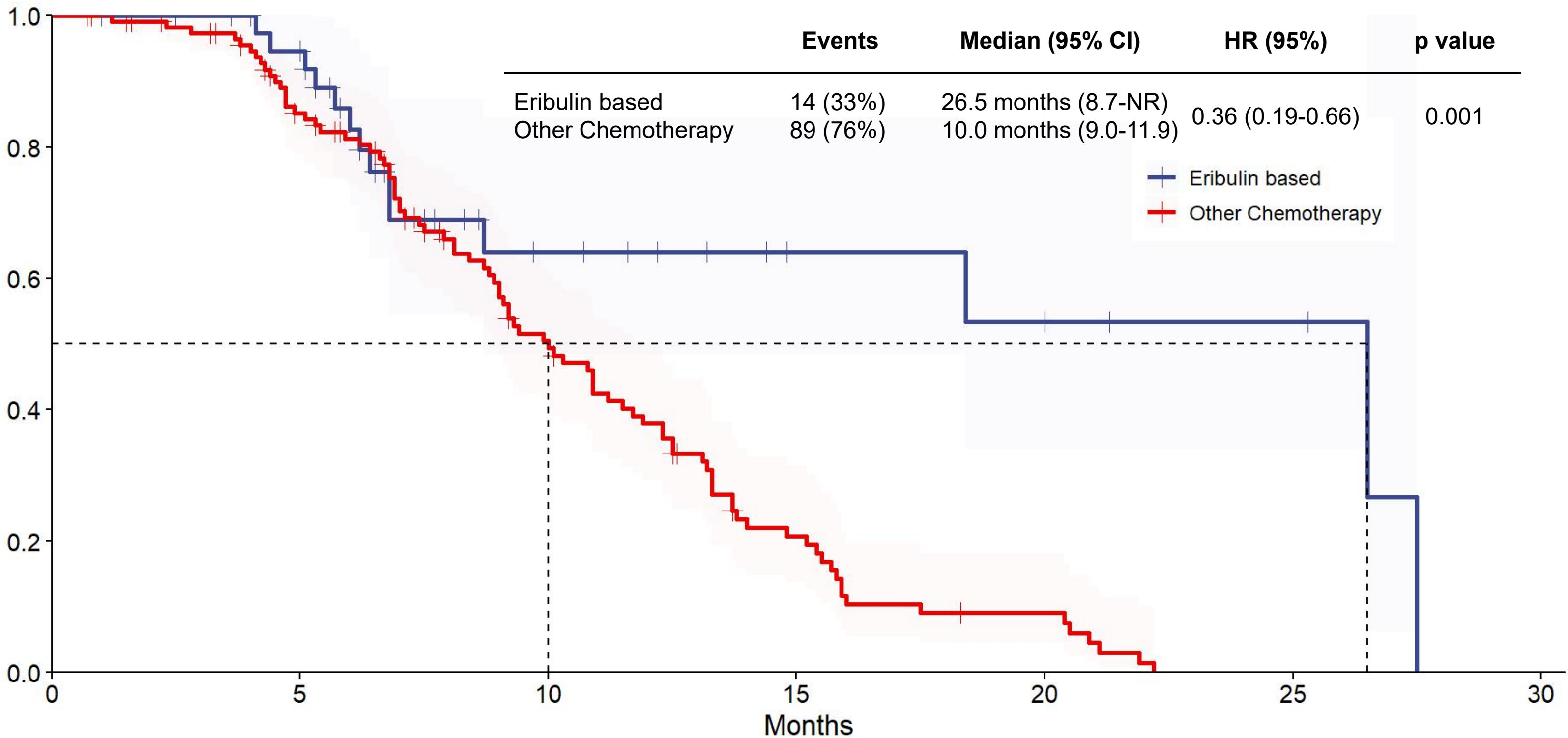

Number at risk

|                    |     |    |    |    |   |   |   |
|--------------------|-----|----|----|----|---|---|---|
| Eribulin based     | 42  | 35 | 12 | 6  | 5 | 3 | 0 |
| Other Chemotherapy | 117 | 89 | 45 | 16 | 6 | 0 | 0 |

Months

Supplement: Supplementary file 2 [file DataSheet2.ZIP › source images/Figure 2 (IJKL). KM Analysis of PFS&OS for Eribulin and Other chemotherapy.pdf]
